# CircRNA-SORE mediates sorafenib resistance in hepatocellular carcinoma by stabilizing YBX1

**DOI:** 10.1038/s41392-020-00375-5

**Published:** 2020-12-26

**Authors:** Junjie Xu, Lin Ji, Yuelong Liang, Zhe Wan, Wei Zheng, Xiaomin Song, Kirill Gorshkov, Qiming Sun, Hui Lin, Xueyong Zheng, Jiang Chen, Ren-an Jin, Xiao Liang, Xiujun Cai

**Affiliations:** 1 Key Laboratory of Laparoscopic Technology of Zhejiang Province, Department of General Surgery, Sir Run-Run Shaw Hospital, Zhejiang University School of Medicine, 310016 Hangzhou, China; 2 Zhejiang University Cancer Center, 310016 Hangzhou, China; 3 Zhejiang Minimal Invasive Diagnosis and Treantment Thechnology Research Center of Severe Hepatobiliary Disease, 310016 Hangzhou, China; 4 National Center for Advancing Translational Sciences, National Institutes of Health, 9800 Medical Center Drive, Bethesda, MD 20892 USA; 5 State Key Laboratory of Molecular Biology, CAS Center for Excellence in Molecular Cell Science, Institute of Biochemistry and Cell Biology, Chinese Academy of Sciences, 200031 Shanghai, China; 6 Department of Biochemistry, and Department of Cardiology of the Second Affiliated Hospital, Zhejiang University School of Medicine, 310058 Hangzhou, China

**Keywords:** Gastrointestinal cancer, Drug development

## Abstract

Sorafenib is the first-line chemotherapeutic therapy for advanced hepatocellular carcinoma (HCC). However, sorafenib resistance significantly limits its therapeutic efficacy, and the mechanisms underlying resistance have not been fully clarified. Here we report that a circular RNA, circRNA-SORE (a circular RNA upregulated in sorafenib-resistant HCC cells), plays a significant role in sorafenib resistance in HCC. We found that circRNA-SORE is upregulated in sorafenib-resistant HCC cells and depletion of circRNA-SORE substantially increases the cell-killing ability of sorafenib. Further studies revealed that circRNA-SORE binds the master oncogenic protein YBX1 in the cytoplasm, which prevents YBX1 nuclear interaction with the E3 ubiquitin ligase PRP19 and thus blocks PRP19-mediated YBX1 degradation. Moreover, our in vitro and in vivo results suggest that circRNA-SORE is transported by exosomes to spread sorafenib resistance among HCC cells. Using different HCC mouse models, we demonstrated that silencing circRNA-SORE by injection of siRNA could substantially overcome sorafenib resistance. Our study provides a proof-of-concept demonstration for a potential strategy to overcome sorafenib resistance in HCC patients by targeting circRNA-SORE or YBX1.

## Introduction

Hepatocellular carcinoma (HCC) is the most common primary liver tumor with an increasing global incidence. In 2018, HCC was the sixth most frequently diagnosed cancer and the fourth leading cause of cancer-related death worldwide.^1^ Sorafenib is the first FDA-approved targeted therapy for advanced HCC. A previous study showed that sorafenib prolonged the median overall survival (OS) by 2.3−3 months in advanced HCC patients that did not qualify for liver transplantation or resection.^2^ However, many HCC patients respond poorly to sorafenib or develop resistance after months of treatment.^3^ Sorafenib resistance in HCC is usually observed within 6 months of treatment.^4^ Compelling evidence has suggested that the primary and acquired resistance of sorafenib in HCC involves multiple mechanisms, including autophagy, epithelial−mesenchymal transition, cancer stem cells, tumor microenvironment, and epigenetic regulation, and a number of signaling pathways are suggested to be involved, such as Wnt/β-catenin, TGFβ, Ras/MEK/ERK, PI3K/Akt, TNFα/NF-κB, and JAK/STAT pathways.^4^ However, the mechanisms of sorafenib resistance, particularly in vivo and in HCC patients, have not been well studied and remain poorly understood.

Circular RNAs (circRNAs) are a class of endogenous noncoding RNAs (ncRNAs) that show complex tissue- and stage-specific expression within the eukaryotic transcriptome.^5^ Compared with linear ncRNAs, circRNAs are much more stable because of their closed-loop structure. Increasing evidence has indicated roles of circRNAs in various cancer-related processes, such as tumor formation, progression, relapse, and drug resistance. One of the best-known functions of circRNA is serving as miRNA sponges by directly binding mRNAs. For example, ciRS-7, the first extensively characterized circRNA, binds miR-7 and acts as a specific miR-7 inhibitor to regulate genes downstream of miR-7 in various cancers.^6,7^ Recent studies have demonstrated that circRNAs can also function by interacting with proteins. For example, circRNA-FOXO3 was shown to inhibit cell cycle progression by forming ternary complexes with p21 and CDK2.^8^

Exosomes are a class of extracellular vesicles that play vital roles in numerous physiological and pathological processes, such as the maintenance of normal physiology, regulation of immune responses, and stimulation of tumor progression.^9,10^ Tumor-derived exosomes have important and fundamental roles in tumor progression.^11^ Recent studies have shown that tumor-derived exosomes may function as transmitters of drug resistance in different malignancies, including HCC.^12,13^ Moreover, circRNAs are enriched in exosomes in some cancers and could thus be used as a class of exosome-based cancer biomarkers.^14^ However, whether and how exosome-transmitted circRNAs are involved in cancer, especially in HCC, remain largely unknown.

In this work, we found that circRNA_104797, which we renamed as circRNA-SORE (a circular RNA upregulated in sorafenib-resistant HCC cells), is critical for maintenance and spread of sorafenib resistance in HCC. We found that circRNA-SORE is upregulated in sorafenib-resistant HCC cells and silencing circRNA-SORE substantially increases sorafenib-induced apoptosis in HCC cells. Mechanistic studies revealed that circRNA-SORE interacts with YBX1 in the cytoplasm, thus preventing PRP19-mediated YBX1 ubiquitination and degradation in the nucleus. Moreover, we found that circRNA-SORE is transmitted by exosomes, and silencing circRNA-SORE in different mouse models leads to a significant increase of sorafenib efficacy.

## Results

### CircRNA-SORE is overexpressed in sorafenib-resistant HCC cells

**Fig. 1:**
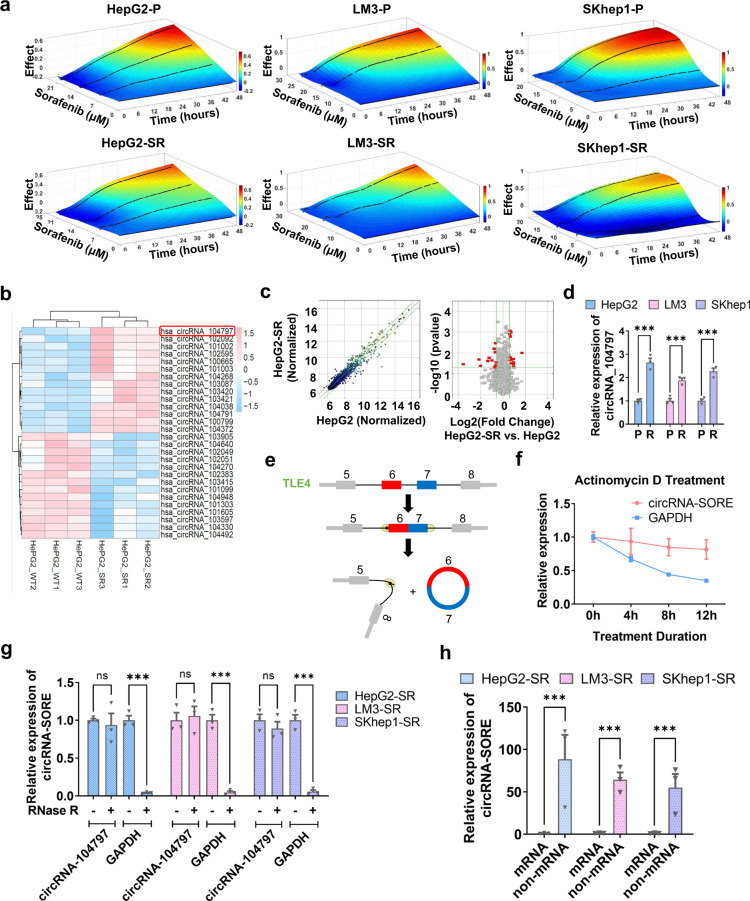
CircRNA-SORE is overexpressed in sorafenib-resistant HCC cells. **a** Three sorafenib-resistant HCC cell lines (HepG2-SR, LM3-SR, and SKhep1-SR) were established and confirmed by the Real-time Cell Analysis xCELLigence System (Roche Applied Sciences). Curves demonstrate the inhibitory effect of sorafenib on parental (P) and sorafenib-resistant (SR) cell lines. **b** Hierarchical clustering of 14 upregulated circRNAs and 14 downregulated circRNAs in HepG2-SR cells compared with parental HepG2 cells by Arraystar Human CircRNA Array analysis. CircRNAs with fold change >2 and *p* < 0.05 were selected as significantly different. **c** Scatter plots (left panel) and volcano plots (right panel) of the differentially expressed circRNAs. Red dots indicate differentially expressed circRNAs (*p* < 0.05 and fold change > 2). **d** CircRNA_104797 levels (normalized by GAPDH) in parental (P) and resistant (R) cell lines treated with the indicated concentrations of sorafenib (7 μM for HepG2, 6 μM for LM3 and SKhep1) for 72 h. **e** Diagram illustrating back-splicing of circRNA-SORE from the *TLE4* host gene. **f** Time-course of the relative expression of circRNA-SORE and *TLE4* in HepG2-SR cells treated with actinomycin D (10 µg/mL). **g** qPCR analysis of circRNA-SORE and GAPDH mRNA in HepG2-SR, LM3-SR and SKhep1-SR cells with or without RNase R treatment for 30 min at 37 °C. **h** Relative expression of circRNA-SORE in mRNA and non-mRNA samples in the indicated cell lines. Three independent experiments with three technical repetitions were performed. Data are expressed as mean ± SEM (error bars). Statistical analyses used Student’s *t* test. ***p < 0.001. NS not significant

Increasing evidence has indicated the involvement of circRNAs in various cancer-related processes. To examine possible circRNAs involved in mediating sorafenib resistance in HCC, we first established three sorafenib-resistant HCC cell lines (HepG2-SR, LM3-SR, and SKhep1-SR) by culturing cells with escalating doses of sorafenib (1–7 μM for HepG2-SR cells and 1–6 μM for LM3-SR and SKhep1 cells) for 12 months. We then confirmed sorafenib resistance in these three cell lines using the Real-time Cell Analysis xCELLigence System. Our results showed that the three sorafenib-resistant cell lines were insensitive to sorafenib and exhibited a faster growth rate under sorafenib treatment compared with their parental cell lines (Fig. 1a). Next, we conducted an Arraystar Human CircRNA Array analysis on parental and HepG2-SR cells as they showed most distinguished difference between parental and resistant cells, and identified 14 significantly upregulated and 14 downregulated circRNAs (circRNAs with fold change > 2 and *p* values < 0.05) in HepG2-SR cells compared with parental control cells (Fig. 1b, c). Among the 14 upregulated circRNAs, circRNA_104797 had the most impact on sorafenib resistance in HCC cells. qPCR analysis confirmed that circRNA_104797 was consistently increased in all three sorafenib-resistant cell lines (Fig. 1d). Thus, we renamed circRNA_104797 as circRNA-SORE (a circular RNA upregulated in sorafenib-resistant HCC cells).

To identify the origin of circRNA-SORE, we checked the UCSC Genome Browser and found that circRNA-SORE (also named circRNA_104797 or circ_0087293) is generated from the back-splicing of the 7th and 8th exons of the *TLE4* gene (Fig. 1e). To verify whether circRNA-SORE has a covalently closed continuous loop structure, we treated cells with actinomycin D (an RNA synthesis inhibitor) and found that circRNA-SORE was more stable than *GAPDH* (Fig. 1f). CircRNA-SORE was also resistant to RNase R, a linear RNA degrader, whereas the control linear GAPDH mRNA was not (Fig. 1g). Moreover, circRNA-SORE was mostly detected in non-mRNA samples (without a poly-A tail) rather than in mRNA samples (with a poly-A tail) (Fig. 1h), indicating that circRNA-SORE has no poly-A tail.

### CircRNA-SORE is critical for maintaining sorafenib resistance

To investigate the possible role of circRNA-SORE in sorafenib resistance, we designed two siRNAs targeting the junction region of circRNA_104797. We found that circRNA-SORE siRNA-1 yielded a better silencing effect (Supplementary Fig. 1A, B), and thus we used siRNA-1 in our subsequent studies. The knockdown efficiency of circRNA-SORE siRNA-1 in all three sorafenib-resistant cell lines was confirmed (Supplementary Fig. 1C).

**Fig. 2:**
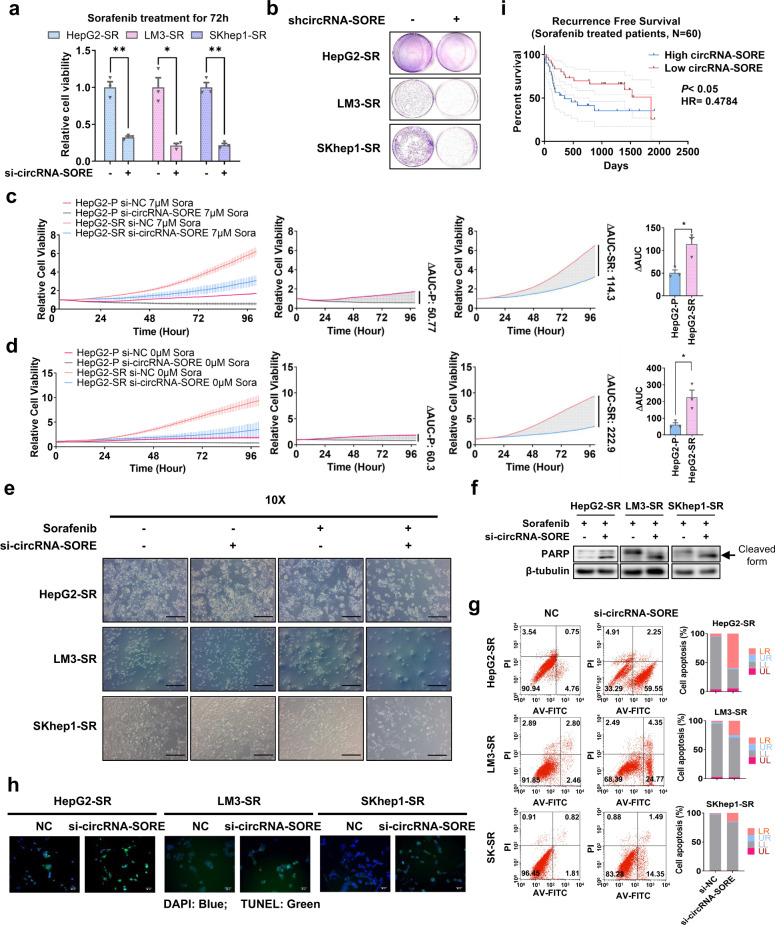
CircRNA-SORE is critical for maintaining sorafenib resistance. **a** Relative cell viability of three HCC sorafenib-resistant cell lines with circRNA-SORE siRNA knockdown (si-circRNA-SORE) compared with controls treated with the indicated concentrations of sorafenib (7 μM for HepG2-SR, 6 μM for LM3-SR and SKhep1-SR) for 72 h. **b** Colony formation assays in three HCC sorafenib-resistant cell lines with or without shcircRNA-SORE. Cells were all treated with sorafenib (7 μM for HepG2-SR, 6 μM for LM3-SR and SKhep1-SR) for 24 h in complete media, washed with PBS and cultured in complete media for another 7 days. **c** HepG2-P and HepG2-SR cell lines transfected with si-circRNA-SORE or negative control siRNA (si-NC) were treated with sorafenib. Cell viability was recorded by Real-time Cell Analysis xCELLigence System and three technical repetitions were performed. ΔAUC (difference of area under curve) = (AUC NC) – (AUC si-SORE). Each technical repetition yields a ΔAUC, and *t* tests were performed. **d** Similar to (**c**) except no sorafenib was added. **e** Brightfield images (objective magnification = 10) showing cell morphology of three sorafenib-resistant cell lines with or without si-circRNA-SORE or sorafenib at the indicated concentrations (7 μM for HepG2-SR, 6 μM for LM3-SR and SKhep1-SR) for 72 h. Scale bar, 20 µm. **f** Western blot analysis of sorafenib-resistant cell lines transfected with si-circRNA-SORE using PARP antibody (upper) and β-tubulin antibody (lower). **g** PI/Annexin V flow cytometry analysis (10,000 cells) of sorafenib-resistant cells transfected with si-NC or si-circRNA-SORE. **h** TUNEL staining (green) of three sorafenib-resistant cell lines transfected with si-NC or si-circRNA-SORE. Nuclei were stained with DAPI (blue). Scale bar, 20 µm. **i** Kaplan−Meier recurrence-free survival analysis for sorafenib-treated HCC patients with low and high circRNA-SORE expression. The median circRNA-SORE expression was used as cut-off for high- and low-expression groups. Three independent experiments with three technical repetitions were performed. Data are expressed as mean ± SEM (error bars). Statistical analyses used Student’s *t* test and Kaplan−Meier survival analysis. *p < 0.05, and **p < 0.01

We next examined the effect of silencing circRNA-SORE on sorafenib resistance in HCC cells. As shown in Fig. 2a, depletion of circRNA-SORE by RNAi remarkably reversed sorafenib resistance in all three sorafenib-resistant HCC cell lines as reflected by cell viability assays. Colony formation assay produced similar results (Fig. 2b). These findings indicate an important role of circRNA-SORE in sorafenib resistance in HCC. To further confirm the function of circRNA-SORE in sorafenib resistance, real-time cell analyses were performed and area under the curve (ΔAUC) was calculated. We found that knocking down circRNA-SORE in HCC cells led to remarkable cell growth inhibitory effects in sorafenib-resistant HCC cells but had less of an impact on parental cell lines (Fig. 2c, d), further confirming a specific role of circRNA-SORE in the maintenance of sorafenib resistance.

Of note, after sorafenib treatment, the cell morphology of all three sorafenib-resistant cell lines transfected with si-circRNA-SORE exhibited signs of apoptosis, but these effects were not observed in the absence of sorafenib treatment or in parental cells (Fig. 2e and Supplementary Fig. S1D). Western blot assays revealed increased poly ADP-ribose polymerase (PARP) cleavage in all three sorafenib-resistant cell lines transfected with si-circRNA-SORE (Fig. 2f), indicating that silencing circRNA-SORE increases sorafenib-induced apoptosis in HCC cells. Propidium iodide/Annexin V apoptosis assays and TUNEL assays further confirmed the marked increase of apoptotic cells in the si-circRNA-SORE-transfected sorafenib-resistant cell lines (Fig. 2g, h and Supplementary Fig. 1E). Conversely, overexpressing circRNA-SORE in all three parental HCC cell lines using pLCDH-ciR-SORE increased cell viability in response to sorafenib treatment (Supplementary Fig. 1F, G). Colony formation assays and propidium iodide/Annexin V apoptosis assays similarly showed that HCC parental cells acquired sorafenib resistance after circRNA-SORE overexpression (Supplementary Fig. 1H, I). Together these results demonstrate that circRNA-SORE plays a critical role in HCC resistance to sorafenib, and silencing circRNA-SORE substantially increases sorafenib-induced tumor cell apoptosis.

Considering the significance of circRNA-SORE in sorafenib resistance in HCC, we then considered whether circRNA-SORE could be a potential predictive marker for sorafenib efficacy in HCC patients. To investigate this possibility, we analyzed a group of 60 sorafenib-treated HCC patients who received liver resection and were treated with sorafenib after surgery from 2009 to 2013 (Table 1). We found that patients with lower circRNA-SORE expression were associated with better recurrence-free survival (RFS) (Fig. 2i and Table 2). This result suggests the potential of circRNA-SORE as a biomarker to predict the efficacy of sorafenib in HCC patients.

**Table 1:** CircRNA-SORE expression and clinicopathological factors in HCC patients

Factors	Low circRNA-SORE expression (95% CI) (*n* = 30) *N* (%)	High circRNA-SORE expression (95% CI) (*n* = 30) *N* (%)	*p* value
Age (mean)	46.77 (42.32−51.21)	48.10 (43.43−52.67)	0.459
Gender			
Male	29 (96.7)	27 (90.0)	
Female	1 (3.3)	3 (10.0)	0.605
HBV			
+	9 (30.0)	12 (40.0)	
−	21 (70.0)	18 (60.0)	0.417
Fibrosis			
+	21 (70.0)	21 (70.0)	
−	9 (30.0)	9 (30.0)	1.000
Tumor size			
(Large) ≥30 mm	28 (93.3)	27 (90.0)	
(Small) <30 mm	2 (6.7)	3 (10.0)	1.000
Tumor number			
>1	5 (16.7)	2 (6.7)	
1	25 (83.3)	28 (93.3)	0.421
Lymph node metastasis			
Present	1 (3.3)	0 (0.0)	
Absent	29 (96.7)	30 (100.0)	1.000
Vascular invasion			
Present	14 (46.7)	11 (36.7)	
Absent	16 (53.3)	19 (63.3)	0.432
Macro-vascular invasion			
Present	5 (16.7)	4 (13.3)	
Absent	25 (83.3)	26 (86.7)	1.000
Micro-vascular invasion			
Present	12 (40.0)	11 (36.7)	
Absent	18 (60.0)	19 (63.3)	0.791
Extrahepatic metastasis			
Present	0 (0.0)	1 (3.3)	
Absent	30 (100.0)	29 (96.7)	1.000
Satellite foci			
Present	3 (10.0)	2 (6.7)	
Absent	27 (90.0)	28 (93.3)	1.000
Stage^a^			
Early	22 (73.3)	23 (76.7)	
Late	18 (26.7)	7 (23.3)	0.766
YBX1^b^			
−/+	27 (90.0)	18 (60.0)	
+ +/+++	3 (10.0)	12 (40.0)	**0.007**

The median circRNA-SORE expression was used as cut-off for high- and low-expression groups

^a^Bureau of Medical Administration, National Health and Family Planning Commission of the People’s Republic of China^41^

^b^YBX1 levels were defined by immunohistochemical staining

**Table 2:** Results of univariate and multivariate analysis of clinicopathological factors for recurrence-free survival (Cox proportional hazard model)

Variables	Univariate analysis			Multivariate analysis (circRNA-SORE)			Multivariate analysis (YBX1)		
	HR	95% CI	*p* value	HR	95% CI	*p* value	HR	95% CI	*p* value
Age (Old/Young)	0.987	0.958−1.015	0.356						
Gender (Female/Male)	1.641	0.496−5.431	0.417	2.830	0.756−10.591	0.122			
HBV (+/−)	1.661	0.795−3.472	0.177						
Fibrosis (+/−)	2.035	0.869−4.764	0.101	2.648	1.059−6.618	**0.037**	2.034	0.860−4.811	0.106
Stage^a^ (Late/ Early)	1.415	0.626−3.202	0.404	1.938	0.814−4.612	0.135			
Micro-vascular invasion (Present/Absent)	1.597	0.785−3.250	0.196				1.998	0.958−4.169	0.065
circRNA-SORE^b^ (High/ Low)	2.053	0.997−4.227	**0.051**	2.281	1.074−4.845	**0.032**			
YBX1^c^ (++/+++ vs. −/+)	2.563	1.214−5.414	**0.014**				2.972	1.380−6.402	**0.005**

As a positive correlation of YBX1 protein level and circRNA-SORE expression level was observed in Table 1, to avoid interations between factors, the multivariate analyses were performed respectively for circRNA-SORE and YBX1

^a^Bureau of Medical Administration, National Health and Family Planning Commission of the People’s Republic of China^41^

^b^The median circRNA-SORE expression was used as cut-off for high- and low-expression groups

^c^YBX1 levels were defined by immunohistochemical staining

### CircRNA-SORE functions by binding to YBX1 protein

One of the best-known functions of circRNA is to interrupt the mRNA splicing of its host gene.^15^ As exon-derived circRNAs and their host mRNAs are derived from the same pre-mRNA, circRNA can interrupt the splicing of its host mRNA, and the canonical pre-mRNA splicing can compete with the process of exon circularization.^15^ CircRNA-SORE is derived from the 7th and 8th exons of the *TLE4* gene and generated into a circular form by back-splicing.^16^ To test whether circRNA-SORE functions through affecting the mRNA splicing of *TLE4*, we compared *TLE4* mRNA levels between sorafenib-resistant and parental cells, but no significant difference was observed (Supplementary Fig. 2A). Next, we examined the effect of silencing circRNA-SORE on the expression of TLE4. As shown in Supplementary Fig. 2B, C, depletion of circRNA-SORE by siRNA had no apparent effect on either TLE4 mRNA or protein levels. These results indicate that circRNA-SORE may not function through regulating its host gene TLE4 expression to mediate sorafenib resistance in HCC.

Recent studies have shown that circRNA−protein interactions may play critical roles in a variety of diseases.^5,6,15,17,18^ Prediction of circRNA-SORE-binding proteins by the online database Circinteractome (https://circinteractome.irp.nia.nih.gov/) suggested that eIF4A3, FMRP, IMP1, and IMP3 may bind circRNA-SORE. We then carried out RNA pull-down assays, which showed that circRNA-SORE indeed bound FMRP, IMP1, and IMP3, but not to eIF4A3 (Supplementary Fig. 2D). To address the potential role of FMRP, IMP1, and IMP3 in sorafenib resistance in HCC, we silenced these genes using siRNA knockdown (Supplementary Fig. 2E, F). However, silencing of FMRP, IMP1, and IMP3 had no effect on sorafenib resistance (Supplementary Fig. 2G), indicating that these proteins are not involved in the function of circRNA-SORE in sorafenib resistance in HCC.

**Fig. 3:**
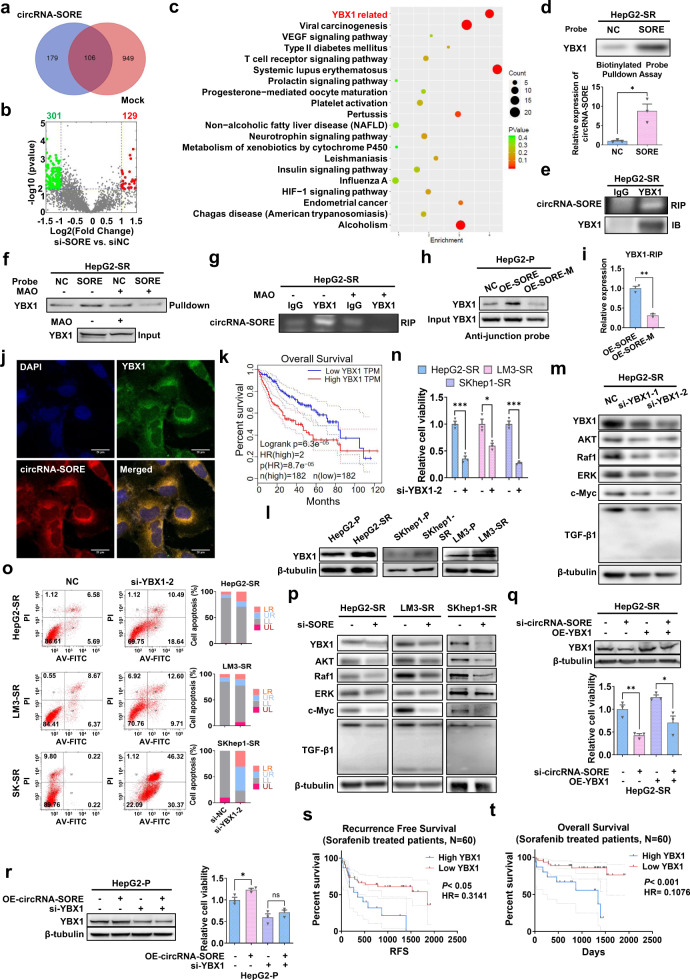
CircRNA-SORE functions by binding to YBX1 protein. **a** Venn diagram illustrating the number of proteins identified using LC-MS/MS from circRNA-SORE pull-down samples in HepG2-SR cells (179 unique proteins, blue) compared with controls (949 unique proteins, red). The overlap (106 proteins, purple) indicates the number of proteins found in both samples. **b** Volcano plot illustrating the 129 upregulated and 301 downregulated proteins of interest (fold change > 2 and *p* < 0.05) in the si-circRNA-SORE group compared with controls in HepG2-SR cells. **c** Bubble diagram of KEGG pathway analysis in si-circRNA-SORE-dysregulated proteins shows the top 20 enriched pathways. **d** Biotinylated-probe pull-down assay for YBX1 using western blot and qPCR analysis for circRNA-SORE in HepG2-SR cells. The probe was designed according to the junction region of circRNA-SORE. **e** Reciprocal RNA immunoprecipitation using YBX1 for circRNA-SORE in HepG2-SR cells. **f** Biotinylated-probe pull-down assay for YBX1 in HepG2-SR cells with or without MAO treatment. The probe was designed according to the junction region of circRNA-SORE. **g** Reciprocal RNA immunoprecipitation using YBX1 for circRNA-SORE in HepG2-SR cells with or without MAO treatment. **h** Biotinylated-probe pull-down assay for YBX1 using western blot in HepG2-P cells overexpressing NC, circRNA-SORE and circRNA-SORE-M. circRNA-SORE-M: circRNA-SORE with a mutated YBX1-binding motif. The probe was designed according to the junction region of circRNA-SORE. **i** RNA immunoprecipitation using YBX1 for circRNA-SORE and circRNA-SORE-M in HepG2-P cells overexpressing circRNA-SORE and circRNA-SORE-M. **j** circRNA-SORE FISH (red), YBX1 immunofluorescence (green), nuclei staining (blue), and merged (yellow) images in HepG2-SR cells. Merged panel shows the colocalization of circRNA-SORE and YBX1. Scale bar, 20 µm. **k** Overall survival curve of HCC patients (*N* = 364) from TCGA database according to high and low YBX1 expression (cut-off: median of YBX1 transcript per million; HR = 2). **l** Western blot analysis of YBX1 and β-tubulin in HCC sorafenib-resistant cells and parental cells. **m** Western blot analysis of YBX1, AKT, Raf1, ERK, c-Myc, TGF-β1 and β-tubulin in HepG2-SR cells transfected with si-NC or YBX1 siRNA. **n** Cell viability of sorafenib-resistant cells transfected with siRNA targeting YBX1 (si-YBX1-2). **o** PI/Annexin V staining and flow cytometry in sorafenib-resistant cells (10,000 cells) following si-NC or si-YBX1-2 transfection. **p** Western blot analysis of YBX1, AKT, Raf1, ERK, c-Myc, TGF-β1, and β-tubulin in sorafenib-resistant cells following si-circRNA-SORE transfection. **q** Western blot analysis of YBX1 and β-tubulin and cell viability in HepG2-SR cells transfected with circRNA-SORE siRNA and overexpressing (OE) YBX1 by lentivirus. **r** Western blot analysis of YBX1 and β-tubulin and cell viability in HepG2-P cells overexpressing (OE) circRNA-SORE by lentivirus and transfected with YBX1 siRNA. **s**, **t** Kaplan−Meier recurrence-free survival analysis and overall survival analysis for sorafenib-treated HCC patients according to low and high YBX1 protein levels. Cases were stratified as low (− and +) and high (++ and + ++) YBX1 protein expression by immunohistochemistry staining. Three independent experiments with three technical repetitions were performed. Data are expressed as mean ± SEM. Statistical analyses used Student’s *t* test. *p < 0.05, **p < 0.01 and ***p < 0.001. Three independent experiments with three technical repetitions were performed. Data are expressed as mean ± SEM (error bars). Statistical analyses used Student’s *t* test, Kaplan−Meier survival analysis, and log-rank test. *p < 0.05, **p < 0.01 and ***p < 0.001

To further investigate potential interacting protein(s) of circRNA-SORE involved in sorafenib resistance, we next carried out LC-MS/MS analysis on circRNA-SORE pull-down samples from HepG2-SR cells and compared it with the controls. The results identified 179 circRNA-SORE-binding proteins (Fig. 3a). To identify proteins regulated by circRNA-SORE, we performed LC-MS/MS analysis on lysates from circRNA-SORE-silenced HepG2-SR cells and compared it with the control cells, which identified 129 upregulated and 301 downregulated proteins (fold change > 2 and *p* < 0.05) (Fig. 3b). Gene Ontology Analysis of the dysregulated proteins in circRNA-SORE-silenced HepG2-SR cells indicated that these proteins are associated with a variety of functions, such as response to drug, cell adhesion and cell migration (Supplementary Fig. 2H). Venn analysis of the circRNA-SORE pull-down proteins and the dysregulated proteins in the circRNA-SORE-silenced group led to the identification of ten overlapping proteins that theoretically should not only bind to but also be regulated by circRNA-SORE (Supplementary Table 2). YBX1, one of the ten candidates, is a nucleic acid-binding protein and more importantly, circRNA-SORE harbors the specific binding sequence (CCAAT) for YBX1.^19^ Moreover, KEGG pathway analysis of si-circRNA-SORE-dysregulated proteins showed a significant enrichment in YBX1-related signaling (*p* < 0.05, Fig. 3c). These results strongly indicated that YBX1 is a binding protein for circRNA-SORE. We then performed RNA pull-down and immunoprecipitation assays and confirmed the specific binding of circRNA-SORE to YBX1 (Fig. 3d, e and Supplementary Fig. 2I, J). To better clarify the interactions between circRNA-SORE and YBX1, morpholino antisense oligos (MAO) targeting the Y-box sequence (CCAAT) in circRNA-SORE were transfected into HepG2-SR cells. As shown in Fig. 3f, g, RNA pull-down and immunoprecipitation assays showed that targeting the Y-box sequence in circRNA-SORE blocked its binding to YBX1, suggesting circRNA-SORE binds YBX1 via the Y-box sequence. Moreover, circRNA-SORE-M, in which the YBX1-binding sequence was mutated, showed reduced binding capacity with YBX1 in RNA pull-down and RIP assays (Fig. 3h, i). CircRNA-SORE FISH and YBX1 immunofluorescence experiments also revealed the colocalization of circRNA-SORE and YBX1 in HepG2-SR cells, which mainly occurred in the cytoplasm (Fig. 3j). These results confirmed an interaction of circRNA-SORE with YBX1.

YBX1 has a well-established role in oncogenesis.^20^ Analysis of RNA sequencing data from The Cancer Genome Atlas (TCGA) project^21^ revealed an increase of YBX1 levels in HCC tumors compared with normal liver tissues (Supplementary Fig. 2K, L). Moreover, survival analysis showed that patients with higher YBX1 levels had worse OS compared with those with lower YBX1 levels (Fig. 3k). Comparison of YBX1 levels between sorafenib-resistant and parental HepG2 cells revealed elevated YBX1 levels in HepG2-SR cells (Fig. 3l), suggesting a role for YBX1 in sorafenib resistance. To further examine the role of YBX1, we silenced YBX1 using two sets of siRNAs, which both remarkably decreased protein levels of YBX1 and its downstream target genes including AKT, Raf1, ERK, c-Myc, and TGF-β1 (Fig. 3m). si-YBX1-2, which showed better knockdown efficiency, was used for subsequent experiments. We next examined the effect of silencing YBX1 on HCC resistance to sorafenib. We found that knocking down YBX1 resulted in attenuated sorafenib resistance, increased apoptosis, and disrupted cell morphology in all three sorafenib-resistant HCC cell lines (Fig. 3o and Supplementary Fig. 2M). Notably, silencing circRNA-SORE markedly decreased the protein levels of YBX1 and its downstream target genes AKT, Raf1, ERK, c-Myc, and TGF-β1 in the three sorafenib-resistant cell lines (Fig. 3p), and the effect of silencing circRNA-SORE on HCC sensitivity to sorafenib could be, to a large extent, rescued by overexpressing YBX1 (Fig. 3q). Importantly, the effect of overexpressing circRNA-SORE on sorafenib resistance was attenuated upon silencing YBX1 in HCC cells (Fig. 3r). Moreover, disrupting the binding of YBX1 and circRNA-SORE using MAO-YBX1 could partially reverse the increase of YBX1 by overexpressing circRNA-SORE in HepG2-P cells (Supplementary Fig. 2N). Together, these results suggest that circRNA-SORE mediates sorafenib resistance in HCC by binding to YBX1.

Finally, we examined YBX1 levels in the 60 sorafenib-treated HCC patients and found that patients with lower YBX1 levels were associated with better RFS (Fig. 3s and Table 2) and better OS (Fig. 3t and Supplementary Table 3). Moreover, in these HCC clinical samples, we observed a positive correlation of YBX1 protein level with circRNA-SORE expression (Supplementary Fig. 2N and Table 1). These results further support a correlation between circRNA-SORE and YBX1 and also suggest that YBX1, similar to circRNA-SORE, may also be used as a biomarker for predicting sorafenib efficacy in HCC patients.

### CircRNA-SORE stabilizes YBX1 by preventing its PRP19-mediated degradation

**Fig. 4:**
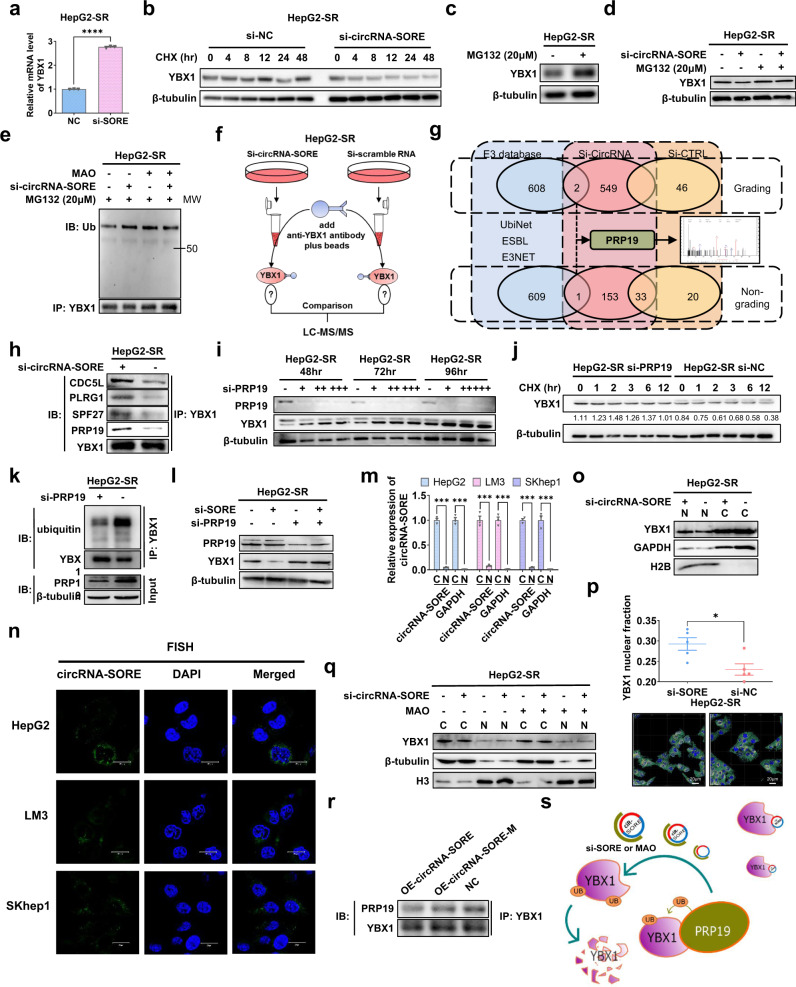
CircRNA-SORE stabilizes YBX1 by preventing its PRP19-mediated degradation. **a** qPCR analysis of YBX1 mRNA expression in HepG2-SR cells transfected with si-NC or si-circRNA-SORE. **b** Western blot analysis of YBX1 and β-tubulin in HepG2-SR cells transfected with si-NC or si-circRNA-SORE and treated with cycloheximide (CHX) (200 μM). **c**, **d** Western blot analysis of YBX1 and β-tubulin in HepG2-SR cells transfected with or without si-circRNA-SORE following proteasome inhibition with MG132 treatment (20 μM) for 3 h. **e** YBX1 was immunoprecipitated (IP) from HepG2-SR cells transfected with or without si-circRNA-SORE and with or without MAO treatment and treated with MG132 (20 μM) for 3 h. Immunoblotting (IB) was performed for ubiquitin (top) and YBX1 (bottom). **f** Schematic illustration of mass spectrometric analysis of YBX1 immunoprecipitation. **g** YBX1 was immunoprecipitated (IP) from HepG2-SR cells transfected with si-NC (orange) or si-circRNA-SORE (blue). Venn diagram illustrating the number of proteins identified using grading and non-grading LC-MS/MS from the YBX1-immunoprecipitated cell lysates. Overlap indicates the number of proteins found in both samples. Purple circle indicates the E3 ligases from the E3 ligase databases (UbiNet, ESBL, and E3NET). The spectrogram of PRP19 from LC-MS/MS is shown on the right. **h** Western blot analysis of YBX1-immunoprecipitated proteins (CDC5L, PLRG1, SPF27 and PRP19) in HepG2-SR cells transfected with or without si-circRNA-SORE. **i** Western blot analysis of YBX1 and β-tubulin in HepG2-SR cells transfected with increasing amounts of siRNA targeting PRP19 (si-PRP19) for 48, 72 and 96 h. **j** Western blot analysis for YBX1 and β-tubulin in HepG2-SR cells transfected with si-PRP19 or si-NC and treated with cycloheximide (CHX) (200 μM). **k** YBX1 was immunoprecipitated (IP) from HepG2-SR cells transfected with the indicated siRNAs and treated with MG132 (20 μM) for 3 h. Immunoblot (IB) was performed with the indicated antibodies. **l** Western blot analysis of PRP19, YBX1 and β-tubulin in HepG2-SR cells transfected with or without si-circRNA-SORE or si-PRP19. **m** Relative expression of circRNA-SORE and GAPDH mRNA in the cytoplasmic (C) and nuclear (N) fractions in the indicated cell lines. **n** Localization of circRNA-SORE (green) was examined in HCC cell lines using FISH. Nuclei are shown in blue. Scale bar, 20 µm. **o** Western blot analysis of YBX1, GAPDH and H2B in cytoplasmic (C) and nuclear (N) fractions in HepG2-SR cells transfected with or without si-circRNA-SORE. **p** Top: the average YBX1 nuclear fractions in HepG2-SR cells transfected with si-circRNA-SORE or si-NC from five different confocal microscopic fields of view. Bottom: merged images of YBX1 immunofluorescence (green) and nuclei staining (DAPI, blue) in HepG2-SR cells transfected with si-circRNA-SORE or si-NC. Yellow (merged signal) indicates the nuclear fraction of YBX1 in HepG2-SR cells. Scale bar, 20 µm. **q** Western blot analysis of YBX1, β-tubulin and H3 in the cytoplasmic (C) and nuclear (N) fractions in HepG2-SR cells transfected with or without si-circRNA-SORE and with or without MAO treatment. **r** Western blot analysis of PRP19 immunoprecipitated by YBX1 in HepG2-P cells transduced with pLCDH-circRNA-ciR (NC), pLCDH-circRNA-SORE (OE-circRNA-SORE), or pLCDH-circRNA-SORE-M (OE-circRNA-SORE-M, with mutant YBX1-binding sites). **s** Schematic of the proposed model. CircRNA-SORE binds to YBX1 in the cytoplasm, thus preventing YBX1 from translocating to the nucleus to interact with and subsequently be degraded by PRP19. Three independent experiments with three technical repetitions were performed. Data are expressed as mean ± SEM (error bars). Statistical analyses used Student’s *t* test. *p < 0.05, ***p < 0.001 and ****p < 0.0001

Our above results showed that silencing circRNA-SORE remarkably decreased the protein levels of YBX1; however, the detailed underlying mechanisms were not clear. Silencing circRNA-SORE resulted in increased mRNA levels of YBX1 (Fig. 4a), suggesting that transcriptional regulation might not be involved in the decrease of YBX1 protein levels. We considered whether circRNA-SORE functions by stabilizing YBX1. To address this possibility, we used cycloheximide (CHX) chase assays and found that YBX1 protein stability was decreased upon circRNA-SORE silencing (Fig. 4b), while treatment with the proteasome inhibitor MG132 increased YBX1 protein levels (Fig. 4c). Importantly, silencing circRNA-SORE did not decrease YBX1 protein levels in HepG2-SR cells treated with MG132 compared with controls without MG132 treatment (Fig. 4d). These results indicate that circRNA-SORE may stabilize YBX1 by preventing its proteasomal degradation.

To examine the possible effect of circRNA-SORE on YBX1 ubiquitination and degradation, we performed immunoprecipitation of YBX1 in HepG2-SR cells with and without silencing of circRNA-SORE and examined its ubiquitination level. As shown in Fig. 4e, silencing circRNA-SORE led to a higher ubiquitination level of YBX1, indicating that circRNA-SORE stabilizes YBX1 by suppressing its ubiquitination. Intriguingly, the si-circRNA-SORE-induced YBX1 ubiquitination was rescued by MAO targeting the Y-box sequence in circRNA-SORE (Fig. 4e), suggesting that circRNA-SORE regulates the ubiquitination of YBX1 through binding to the YBX1-binding site.

To identify the potential E3 ubiquitin ligase involved in circRNA-SORE-mediated YBX1 stabilization, we performed mass spectrometric analysis of YBX1-immunoprecipitated complexes from HepG2-SR cells transfected with si-circRNA-SORE compared with controls (Fig. 4f). We cross-referenced the results with the E3 ubiquitin ligase databases (UbiNet, ESBL, and E3NET) and found that two E3 ligases, Pre-MRNA Processing Factor 19 (PRP19) and Notchless protein homolog 1 (NLE1), were identified exclusively in the si-circRNA-SORE-transfected cells by grading mass spectrum, but only PRP19 was also identified by non-grading mass spectrum in si-circRNA-SORE-transfected cells (Fig. 4g). In addition, other components of the PRP19 complex (also known as the NTC, nineteen complex), including CDC5L, PLRG1, and SPF27, were also found exclusively in YBX1-immunoprecipitated complexes from lysates of si-circRNA-SORE-transfected cells (Fig. 4h).

PRP19 complex, a ubiquitin ligase, mediates protein degradation through the ubiquitin proteasome pathway.^22^ To confirm whether PRP19 is the E3 ligase for YBX1, we first tested the effect of silencing PRP19 on YBX protein level. We found that YBX1 protein level was increased in HepG2-SR cells transfected with siRNA targeting PRP19 in a concentration- and time-dependent manner (Fig. 4i). These results indicate that PRP19 mediates YBX1 degradation. Consistent with this result, CHX chase assays showed that silencing PRP19 could stabilize YBX1 protein levels (Fig. 4j). Moreover, the ubiquitination of YBX1 was remarkably decreased in HepG2-SR cells upon PRP19 silencing (Fig. 4k). In particular, silencing PRP19 rescued the decrease of YBX1 mediated by si-circRNA-SORE (Fig. 4l). Together, these results suggest that PRP19-mediated YBX1 ubiquitination and degradation is involved in the circRNA-SORE-YBX1 regulation.

Next, we examined the localization of circRNA-SORE, PRP19 and YBX1. As shown in Fig. 4m, circRNA-SORE, similar to GAPDH mRNA, was primarily detected in the cytoplasm of HCC cells rather than in the nucleus. Fluorescence in situ hybridization (FISH) for circRNA-SORE further confirmed its cytoplasmic localization in the three HCC cell lines (Fig. 4n). More importantly, we found that YBX1 was partially translocated from the cytoplasm to the nucleus upon transfection with si-circRNA-SORE (Fig. 4o). Consistent with this finding, immunofluorescence experiments also showed enhanced nuclear localization of YBX1 in cells transfected with si-circRNA-SORE compared with the controls (Fig. 4p). Notably, a previous study reported that PRP19 is located in the nucleus.^23^ These results suggest that circRNA-SORE may prevent YBX1 from translocating into the nucleus, where it is ubiquitinated and degraded by PRP19.

To further confirm the inhibitory effect of circRNA-SORE on the YBX1-PRP19 interaction, MAOs targeting the Y-box motif were transfected in HepG2-SR cells. As shown in Fig. 4q, MAOs partially rescued the nuclear translocation of YBX1 upon treatment of si-circRNA-SORE. Additionally, overexpression of wild-type circRNA-SORE decreased the interaction between PRP19 and YBX1, whereas overexpression of the mutant circRNA-SORE had no effect (Fig. 4r). Based on these results, we proposed a model in which circRNA-SORE binds to YBX1 in the cytoplasm, thus preventing YBX1 from translocating to the nucleus to interact with and subsequently be degraded by PRP19 (Fig. 4s).

### CircRNA-SORE transmits sorafenib resistance by exosomes

**Fig. 5:**
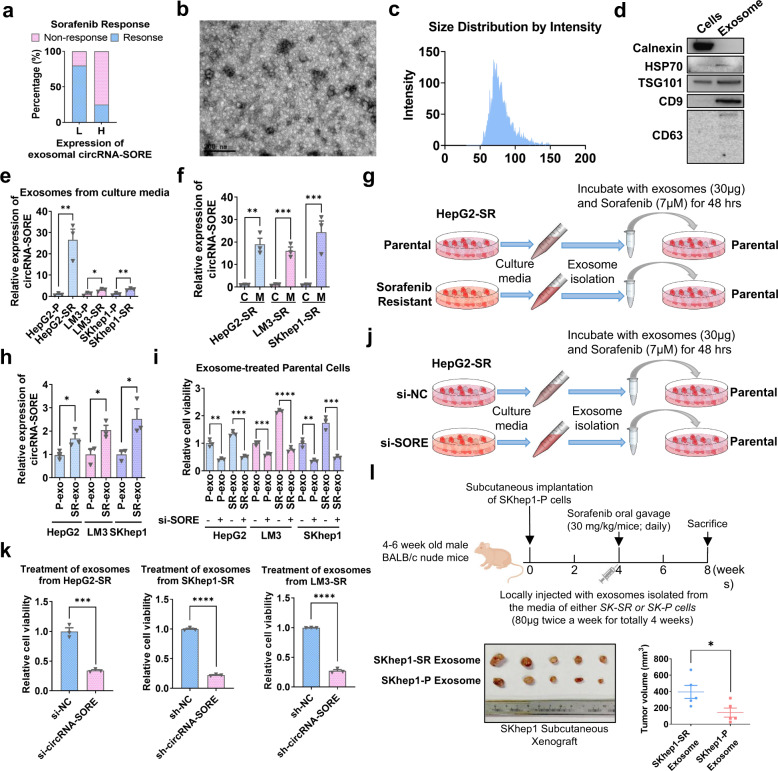
CircRNA-SORE transmits sorafenib resistance by exosomes. **a** Exosomes were extracted from the blood of nine HCC patients prior to sorafenib treatment, and exosomal circRNA-SORE expressions were evaluated by qPCR assays. Lower and higher circRNA-SORE expressions were stratified by the average expression among the nine patients. Response to sorafenib was defined as once achieved complete response, partial response or stable disease for >3 months. **b** Negative staining electron micrograph of exosomes isolated from HepG2-SR culture media. Scale bar, 200 nm. **c** Size distribution of exosomes by NanoSight NS300 instrument. **d** Western blot analysis for positive exosome markers (HSP70, TSG101, CD9 and CD63) and negative exosome marker (Calnexin). **e** qPCR analysis of exosomal circRNA-SORE isolated from sorafenib-resistant and parental cell culture media. **f** qPCR analysis of circRNA-SORE isolated from sorafenib-resistant cell culture media (M) and cells (C). **g** Schematic showing that exosomes were isolated from parental and sorafenib-resistant cells (P-exo and SR-exo, respectively) and used to treat (30 μg) parental cells for 24 h. The exosome-treated parental cells were then transfected with si-NC or si-circRNA-SORE and treated with sorafenib for 48 h. **h**, **i** Exosomes were isolated from parental and sorafenib-resistant cells (P-exo and SR-exo, respectively) and used to treat parental cells for 24 h. The exosome-treated parental cells were then transfected with si-NC or si-circRNA-SORE and treated with sorafenib for 48 h. Charts show **h** qPCR results of circRNA-SORE expression and **i** cell viability in exosome-treated parental cells. **j** Schematic showing that HepG2-SR cells were treated with si-NC or si-circRNA-SORE before the culture media were harvested for exosome isolation and subsequent exosome treatments (30 μg) on HepG2 parental cells. **k** HepG2-SR, SKhep1-SR and LM3-SR cells were treated with si-NC or si-circRNA-SORE before the culture media were harvested for exosome isolation and subsequent exosome treatments on parental cells. The chart shows the cell viability in exosome-treated parental cells. **l** BALB/c nude mice (4- to 6-week-old males) were subcutaneously injected with SKhep1-P cells. On week 4, the mice were treated with sorafenib (30 mg/kg/day) by oral gavage and exosomes (80 μg twice a week for 4 weeks) from SKhep1-SR or SKhep1-P cell culture media by injection at the implantation site twice a week for 4 weeks. Animals were sacrificed on week 8 and xenografts were isolated and measured. Three independent experiments with three technical repetitions were performed. Data are expressed as mean ± SEM (error bars). Statistical analyses used Student’s *t* test. *p < 0.05, **p < 0.01, ***p < 0.001 and ****p < 0.0001

Recent studies have demonstrated that circRNAs are enriched in exosomes.^14^ Other reports showed that drug resistance can be transmitted by exosomes.^12,13^ We next evaluated exosomal circRNA-SORE expressions in the blood of nine HCC patients prior to sorafenib treatment. As shown in Fig. 5a, patients with relatively lower circRNA-SORE expression had a higher response rate to sorafenib (80%) compared with patients with higher circRNA-SORE expression (25%), indicating that exosomes might be involved in circRNA-SORE-mediated sorafenib resistance in HCC. To test this possibility, we isolated and identified exosomes from the culture media of HepG2-SR and HepG2-P cells (Fig. 5b–d). qRT-PCR revealed that exosomes isolated from HepG2-SR cell culture media contained more circRNA-SORE than those from HepG2-P cell culture media (Fig. 5e). Moreover, circRNA-SORE was substantially more enriched in exosomes from the culture media of sorafenib-resistant cells than in the cell cytosol (Fig. 5f), further supporting that exosomes are involved in transmitting circRNA-SORE. Furthermore, we found that parental HCC cells treated with exosomes from the sorafenib-resistant cell culture media showed higher levels of circRNA-SORE and were more resistant to sorafenib than cells treated with the exosomes from parental cell culture media (Fig. 5g, h). This effect was partially rescued by silencing circRNA-SORE in exosome-treated parental HCC cells (Fig. 5i). These results suggest a role for exosomes in spreading circRNA-SORE-mediated sorafenib resistance among HCC cells.

In vitro engineered exosomes carrying siRNAs targeting circRNA-SORE represent potential tumor-specific and molecular-targeted therapies in HCC patients.^24^ Novel exosome-based cancer therapeutic strategies have been extensively reported, including the preclinical use of dendritic cell-derived exosomes for HCC immunotherapy^25^ and stellate cell-derived miR-335-5p-loaded exosomes for HCC treatment.^26^ Notably, we found that parental HCC cells treated with exosomes from circRNA-SORE-silenced sorafenib-resistant cells were more sensitive to sorafenib than cells treated with exosomes from control cells (Fig. 5j, k). The targeting nature of exosomes also makes them ideal transmitters for delivering therapeutic agents, such as therapeutic siRNAs.

To further investigate the involvement of exosomes in the function of circRNA-SORE in vivo, we subcutaneously implanted SKhep1-P cells in BALB/c nude mice and then treated mice with sorafenib for 4 weeks (Fig. 5l). Exosomes from the culture media of SKhep1-SR and SKhep1-P cells were then locally injected around the tumor implantation site. After 4 weeks, mice were sacrificed and tumors were harvested. The tumors from mice receiving exosomes from SKhep1-SR cells were significantly larger than those from mice receiving exosomes from SKhep1-P cells (*p* < 0.05), indicating an enhanced drug resistance in the SKhep1-SR exosome-treated group. Together these results demonstrate that exosomes are crucial for spreading circRNA-SORE-mediated sorafenib resistance among HCC tumor cells.

### Silencing circRNA-SORE substantially improves sorafenib efficacy in vivo

**Fig. 6:**
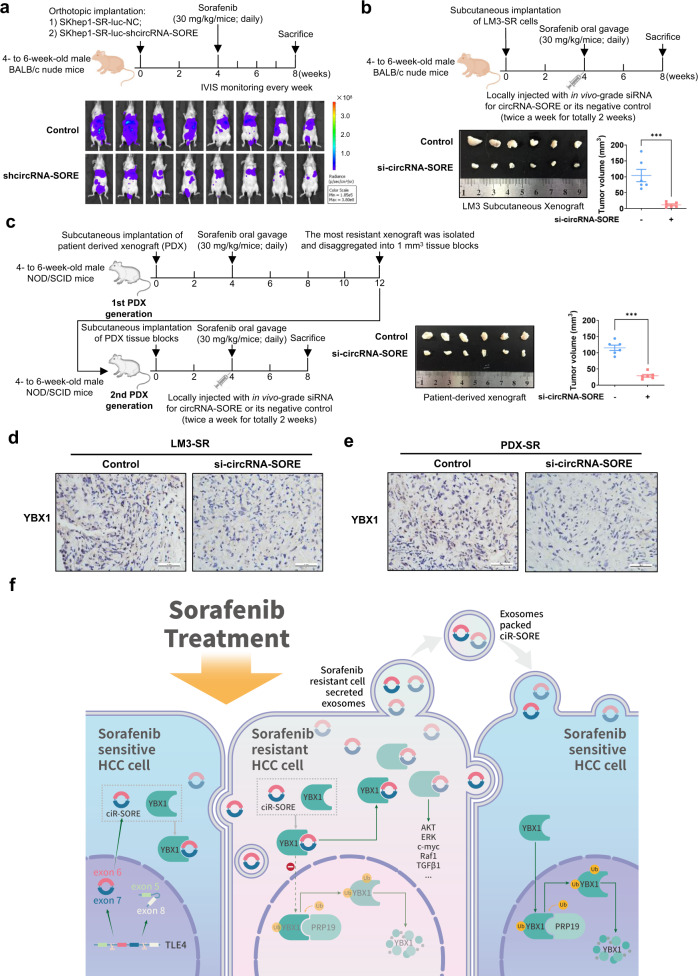
Silencing circRNA-SORE substantially improves sorafenib efficacy in vivo. **a** BALB/c nude mice (4- to 6-week-old males) were orthotopically implanted with SKhep1-SR-luc-NC or SKhep1-SR-luc-shcircRNA-SORE cells. On week 4, the mice were treated with sorafenib (30 mg/kg/day) for 4 weeks. In vivo luminescent imaging was performed weekly starting on week 4. Animals were sacrificed on week 8. Luminescence intensity ranges from low (blue) to high (red). **b** BALB/c nude mice (4- to 6-week-old males) were subcutaneously injected with LM3-SR cells. On week 4, the mice were treated with sorafenib (30 mg/kg/day) for 4 weeks and then injected at the implantation site with in vivo-grade si-circRNA-SORE or si-NC twice a week for 2 weeks. Animals were sacrificed on week 8. **c** NOD/SCID mice (4- to 6-week-old males) were subcutaneously implanted with HCC patient-derived xenograft (PDX) and treated with sorafenib (30 mg/kg/day) by oral gavage starting on week 4. Eight weeks later, the sorafenib-resistant xenograft was removed for a second round of orthotopic implantation. On week 4, the second PDX generation mice were treated with sorafenib (30 mg/kg/day) for 4 weeks and were injected at the implantation site with in vivo-grade si-circRNA-SORE or si-NC twice a week for 2 weeks. Animals were sacrificed and the second-generation PDX was isolated on week 8. **d** Immunohistochemistry for YBX1 in si-circRNA-SORE and si-NC LM3-SR xenografts. Scale bar, 50 µm. **e** Immunohistochemistry for YBX1 in si-circRNA-SORE and control PDX. Scale bar, 50 µm. **f** Model for circRNA-SORE-mediated sorafenib resistance in HCC. CircRNA-SORE is necessary for the development and maintenance of sorafenib resistance in HCC, and silencing circRNA-SORE substantially increases sorafenib-induced apoptosis. CircRNA-SORE specifically binds to the master oncogenic protein YBX1 and thus sequesters YBX1 in the cytoplasm, which prevents YBX1 ubiquitination and degradation by PRP19 in the nucleus. CircRNA-SORE is transmitted by exosomes, allowing for the spread of sorafenib resistance among HCC cells. Data are expressed as mean ± SEM (error bars). Statistical analyses used Student’s *t* test. ***p < 0.001

To further confirm the significance of circRNA-SORE in mediating HCC resistance to sorafenib in vivo, we used three different mouse models: an orthotopic sorafenib-resistant cell line-derived xenograft (CDX) model, a subcutanous sorafenib-resistant CDX model and a sorafenib-resistant patient-derived xenograft (PDX) model. Mice with orthotopic implantation of SKhep1-SR cells expressing shcircRNA-SORE were markedly more sensitive to sorafenib treatment than mice implanted with SKhep1-SR-shNC cells (Fig. 6a). In the second mouse model, we locally injected in vivo grade cholesterol-conjugated RIG-I si-circRNA-SORE around the LM3-SR cell implantation site in BALB/c nude mice, which significantly increased the sensitivity of mice to sorafenib treatment compared with injection of control siRNA (*p* < 0.001, Fig. 6b). In the third model, the sorafenib-resistant PDX model, local injection of in vivo grade cholesterol-conjugated RIG-I si-circRNA-SORE around the PDX implantation site resulted in significant increases in the sensitivity to sorafenib treatment compared with injection of control siRNA (*p* < 0.001, Fig. 6c). Moreover, similar to the in vitro results, YBX1 level was reduced by si-circRNA-SORE treatment, both in the LM3-SR xenograft and the PDX model (Fig. 6d, e). Overall, these results demonstrated the in vivo effect of depriving circRNA-SORE on enhanced sorafenib sensitivity in three animal HCC models, further supporting the clinical potential of silencing circRNA-SORE to improve sorafenib efficacy in HCC patients.

## Discussion

HCC is a highly therapy-resistant cancer and surgery is currently the primary treatment option but is only suitable for HCC patients who are diagnosed at an early stage. However, HCC patients are frequently diagnosed at advanced stages, and the interventions for these patients, including molecular-targeted therapies, are limited and show low efficacy.^27,28^ As the first FDA-approved molecular-targeted drug, sorafenib provided a 3-month prolongation of the median OS time according to The Sorafenib HCC Assessment Randomized Protocol (SHARP) trial^29^ and the Phase III Sorafenib Asia-Pacific (AP) trial.^2^ However, sorafenib resistance, which often develops within 6 months, has caused widespread concerns and significantly limits the clinical benefit of sorafenib in HCC patients. Despite great efforts in the development of combined therapies to improve sorafenib efficacy, the overall outcomes of liver cancer treatment are still far from satisfactory.^27,30–34^ Therefore, there is an urgent need to further explore the mechanisms underlying sorafenib resistance for the development of more effective combination therapies. In this study, we uncovered a previously unrecognized role of a circRNA, circRNA-SORE, in maintaining and transmitting sorafenib resistance in HCC. These results provide a new potential combination therapy strategy to overcome sorafenib resistance in HCC.

CircRNAs, which were identified more than 20 years ago, were initially thought to have low abundance and result from alternative splicing errors during transcription. Based on high-throughput sequencing and novel computational approaches, circRNAs derived from exons or introns are now recognized as widespread and diverse endogenous eukaryotic ncRNAs that participate in various cancer-related processes.^5,6,35^ CircRNAs have a covalently closed-loop structure with neither 5′ to 3′ polarity nor a polyadenylated tail, which makes circRNAs more stable than the linear counterparts. Moreover, circRNAs contain multiple binding sites to trap specific molecules and thus show more specific and efficient functionality compared with miRNAs. In particular, the tissue- and stage-specific expression of circRNAs makes these molecules suitable therapeutic target candidates. In our study, local injection of in vivo-grade cholesterol-conjugated RIG-I si-circRNA-SORE significantly increased the sensitivity of sorafenib treatment in HCC mouse models, indicating the promising clinical application of siRNA-mediated regulation of circRNA-SORE in HCC patients. Indeed, the U.S. Food and Drug Administration recently approved the first RNAi therapeutic, Onpattro infusion, for the treatment of polyneuropathy, a peripheral nerve disease.^36^ Our findings suggest the promising prospect of in vivo delivery of siRNA targeting circRNA-SORE by transarterial chemoembolization or other means to enhance sorafenib efficacy in HCC patients.

Previous studies reported that exon-derived circRNAs are located predominantly in the cytoplasm.^37^ Consistent with this study, we also found that circRNA-SORE is predominantly located in the cytoplasm of HCC cells. Moreover, we identified YBX1 as the binding partner for circRNA-SORE in the cytoplasm. YBX1, also known as YB-1, is highly correlated with tumor cell proliferation, drug resistance, cancer progression, and prognosis in various types of cancer.^20^ Our results showed that circRNA-SORE binds to YBX1 in the cytoplasm, thus preventing YBX1 nuclear translocation and consequently blocking its PRP19-mediated ubiquitination and degradation. PRP19 contains a U-box domain that is structurally related to the RING finger motif found in certain E3 ubiquitin ligases and exhibits E3 ligase activity. PRP19 functions in protein degradation and may transport its substrates to the proteasome by binding to proteasomal subunits.^38,39^ Although other components of the PRP19 complex were also found exclusively in YBX1-immunoprecipitated complexes from lysates from si-circRNA-SORE-transfected cells, whether PRP19 functions alone or as a part of a complex in mediating protein ubiquitination is unclear. Further studies are required to clarify whether other components of the PRP19 complex are also involved in YBX1 degradation.

Recent studies demonstrated that tumor-derived exosomes could function as transmitters of drug resistance in different malignancies.^12,13^ Moreover, a recent study showed that exon-derived circRNAs were stably enriched in exosomes,^14^ and adipose exosomes carrying circRNAs were found to promote the growth of HCC by targeting USP7.^40^ In line with these findings, here we found that circRNA-SORE is enriched and transmitted by exosomes, enabling the spread of sorafenib resistance among HCC cells. The stable properties of exosomes and circRNAs may make exosome-carrying-circRNA-SORE a promising biomarker in liquid biopsy for the early detection and noninvasive surveillance of HCC.

In conclusion, our results demonstrate that circRNA-SORE plays a critical role in the maintenance and transmission of sorafenib resistance in HCC (Fig. 6f). CircRNA-SORE functions by preventing PRP19-mediated YBX1 degradation, thereby affecting the expression of downstream gene targets of YBX1 including AKT, Raf1, ERK, c-Myc, and TGF-β1. CircRNA-SORE is transported by exosomes, which enables transmission of sorafenib resistance among HCC cells. Local siRNA-mediated silencing of circRNA-SORE effectively overcomes sorafenib resistance and increases therapeutic efficacy in different HCC mouse models. Together, these findings suggest a potential clinical application of circRNA-SORE siRNA in combination with sorafenib to treat patients with advanced HCC. More clinical samples, especially more blood samples from HCC patients before and after sorafenib treatment, are required to fully establish the clinical value of targeting circRNA-SORE or YBX1 for overcoming sorafenib resistance in HCC patients.

## Materials and methods

### RNA pull-down

For biotinylated RNA pull-down assays, transfected HepG2-SR cell lysates were incubated with MyOne™ Dynabead® Streptavidin C1 beads (Invitrogen, USA) at 4 °C for 1 h. For biotinylated-probe pull-down assays, MyOne™ Dynabead® Streptavidin C1 beads were incubated with HepG2-SR cell lysate at 4 °C for 1 h for preclearance. The 3′ biotin-labeled circRNA-SORE probe (5′-GAGTTGTTGCTGCTTGATGGAGTC-3′-biotin) was synthesized by Tsingke Biotech (Beijing, China) and incubated with the beads at room temperature for 10 min for immobilization. The biotinylated beads were incubated with HepG2-SR cell lysates at 4 °C overnight. The beads were then magnetically separated and washed five times. For western blot detection, the beads were boiled in SDS buffer for protein/peptide isolation. For mass spectrum assays, the beads were incubated with non-ionic water in 70 °C for 5 min.

### Silencing and overexpression of circRNA-SORE

CircRNA-SORE siRNA targeting the junction region of the circRNA-SORE sequence was synthesized by RiboBio (Guangzhou, China). The short hairpin shcircRNA-SORE sequence (based on the same sequence as si-circRNA-SORE) was cloned into GV248.

The pLCDH-ciR vector (Geneseed, Guangzhou, China) was used to construct the circRNA-SORE overexpression plasmid (OE-circRNA-SORE). The linear sequence of circRNA-SORE together with the flanking circRNA inducing sequence was amplified and subcloned into *Eco*RI and *Bam*HI sites of pLCDH-ciR, and the resulting vector was termed pLCDH-ciR-SORE. pLCDH-circRNA-SORE-M has a mutated YBX1-binding site. The sequences of mutant constructs are listed in Supplementary Table 1. The primers were as follows:

Primer-F: CGGAATTCTGAAATATGCTATCTTACAGCAGCAACAACTCCAGGCCCA and

Primer-R: CGGGATCCTCAAGAAAAAATATATTCACCTTGATGGAGTCTCTGTCTC.

GV248-shcircRNA-104797, pLCDH-ciR, pLCDH-ciR-104797, psAX2 packaging plasmid, and pMD2G envelope plasmids were transfected into 293T cells using the standard calcium phosphate transfection method for 48 h to produce lentivirus. Viruses were collected from supernatant and concentrated by density gradient centrifugation. Viruses were frozen at −80 °C for later use.

### Tissue samples

For mouse studies, we collected all samples from the NOD/SCID mice and BALB/c nude mice for hematoxylin-eosin staining and immunohistochemistry staining according to the designated antibody protocols. Tissue samples (60 cases) and blood samples (9 cases) were obtained from randomly selected sorafenib-treated HCC patients. All patients signed informed consent for the use of tissues for scientific research. We reviewed pathology records to identify samples with confirmed HCC. Low- and high circRNA-SORE expression groups were classified using median expression as a cut-off value. The current study conformed to the principles of the Declaration of Helsinki and was approved by the Institutional Review Board of the Sir Run-Run Shaw Hospital.

Additional methods information can be found in Supplementary Materials.

## Supplementary information

Supplementary Files

## Data Availability

Additional data collected during this study are available from the corresponding authors upon reasonable request.
