# Supplementary material for: CircRNA-SORE mediates sorafenib resistance in hepatocellular carcinoma by stabilizing YBX1
Source: Signal Transduct Target Ther. 2020 Dec 26;5:298. doi: 10.1038/s41392-020-00375-5 (PMC7762756; doi:10.1038/s41392-020-00375-5)
Supplement: Supplementary file 1 — Supplementary Files [file 41392_2020_375_MOESM1_ESM.pdf]

# Supplementary Materials for

## CircRNA-SORE Mediates Sorafenib Resistance in Hepatocellular Carcinoma by Stabilizing YBX1

Junjie Xu†, Lin Ji†, Yuelong Liang†, Zhe Wan, Wei Zheng, Xiaomin Song, Kirill Gorshkov, Qiming Sun, Hui Lin, Xueyong Zheng, Jiang Chen, Ren-an Jin, Xiao Liang, and Xiujun Cai

Correspondence to: Xiujun Cai (srrsh_cxj@zju.edu.cn) and Xiao Liang (srrshlx@zju.edu.cn).
†These authors contributed equally to this work.

**This PDF file includes:**

Supplementary Materials and Methods
Supplementary Figure Legends
Supplementary Figures. 1 to 2
Supplementary Tables 1 to 3
References

## Materials and Methods

Materials

Sorafenib was purchased from Selleck Chemicals (Houston, TX, USA). Actinomycin D was purchased from Sigma-Aldrich (St. Louis, MO, USA). RNase R was purchased from Epicentre (Madison, WI, USA). MG132 and cycloheximide (CHX) were purchased from MedChemExpress (Monmouth Junction, NJ, USA).

*In vitro* cell culture/maintenance

The human HCC cell lines HepG2 (RRID: CVCL_0027) and SKhep1 (RRID: CVCL_0525) were purchased from the American Type Culture Collection (Manassas, VA, USA). LM3 cells (RRID:CVCL_D269) were purchased from the China Center for Type Culture (Wuhan, Hubei, China). All the cell lines were mycoplasma negative and identified with authentication reports. Mycoplasma Test Kit GMyc-PCR (YEASEN, Shanghai, China) was used for mycoplasma detection. All the cell lines were cultured in Dulbecco's Modified Eagle's Media (Invitrogen, Grand Island, NY, USA) supplemented with 10% FBS (v/v), penicillin (25 units/ml), streptomycin (25 g/ml), 1% L-glutamine, and 10% fetal bovine serum (FBS) in a 5% (v/v) CO2 humidified incubator at 37° C. CCK-8 assays were conducted to confirm the resistance of each cell line to sorafenib. Cell growth curves were automatically recorded on the xCELLigence System (Roche Applied Sciences) in real-time. The cell index was followed for 3 days. For treatment with MAOs, each MAO (GeneTools, Philomath, OR, USA) was added to culture media at a final concentration of 10 μM along with Endoporter (GeneTools, Philomath, OR, USA) and incubated for 24 hours. The sequence of MAOs targeting Y-box motif: ATCTTTAATTGGAAGATGGGACTGA.

Microarray analysis

Arraystar Human circRNA Array analysis specifically for human circular RNAs splicing sites was used with six HCC cell samples (three parental HepG2 samples and three sorafenib resistant HepG2 samples). Total RNA from each sample was quantified using the NanoDrop ND-1000. The sample preparation and microarray hybridization were performed based on the Arraystar's standard protocols. Briefly, total RNA was digested with RNase R (Epicentre, Inc.) to remove linear RNA and enrich circular RNA. Then, the enriched circular RNA was amplified and transcribed into fluorescent cRNA utilizing a random priming method (Arraystar Super RNA Labeling Kit; Arraystar). The labeled cRNAs were hybridized onto the Arraystar Human circRNA Array (8x15K, Arraystar). After washing the slides, the arrays were scanned by the Agilent Scanner G2505C. Agilent Feature Extraction software (version 11.0.1.1) was used to analyze acquired array images. Quantile normalization and subsequent data processing were performed using the R software package. Differentially expressed circRNAs with statistical significance between the two groups were identified through Volcano Plot filtering. Differentially expressed circRNAs between two samples were identified through Fold Change filtering. Hierarchical Clustering was performed to show the distinguishable circRNAs expression pattern among samples. Microarray data have been deposited in NCBI's Gene Expression Omnibus (GEO) database (www.ncbi.nlm.nih.gov/geo) under accession number GSE101850.

Western blot analysis

Cells were lysed in lysis buffer and proteins (30 μg) were separated on 10 – 12% SDS/PAGE gel and then transferred onto PVDF membranes (Millipore, Billerica, MA). After blocking membranes, they were incubated with appropriate dilutions (1:2000 for internal control antibodies and 1:1000 for others) of specific primary antibodies against β -tubulin, YBX1, ubiquitin, PRP19, CDC5L, SPF27, IMP3, FMRP or eIF4A3 (from Abcam, Cambridge, MA), PARP (from Cell Signaling, Danvers, MA), PLRG1 and IMP1 (from Bethyl, Montgomery, TX). The blots were incubated with HRP-conjugated secondary antibodies and visualized using the ECL system (Thermo Fisher Scientific, Rochester, NY).

<u>CCK8 assay and colony formation assay</u>

The viability of HCC cells was determined by Cell Counting Kit 8 (Dojindo, Japan) and measured at OD 450 nm with the BioTek Gen5 system (BioTeck, USA). For colony formation assay, 6-cm dishes were seeded with 1 × 104 viable cells infected with shcircRNA-SORE or control virus and allowed to grow for 24 hours. The cells were then incubated in the presence of sorafenib for 24 hours in complete media, and after wash with PBS gently, the cells were allowed to grow in complete media for another 7 days. The colonies obtained were washed gently with PBS and fixed in 4% paraformaldehyde for 20 minutes at room temperature and then washed with PBS followed by staining with 0.1% (w/v) crystal violet. The colonies with >50 cells under the microscope were counted. Three different independent experiments were performed.

<u>RNA extraction, reverse transcription, and Quantitative Real-Time PCR analysis</u>

For RNA extraction, total RNA was isolated using Trizol reagent (Invitrogen, Grand Island, NY). 1 μg of total RNA was subjected to reverse transcription using Superscript III transcriptase (Invitrogen, Grand Island, NY). Quantitative real-time PCR (qRT-PCR) was conducted using a Bio-Rad CFX96 system with SYBR green to determine the mRNA expression level of a gene of interest. Expression levels were normalized to the expression of β -actin RNA. For circRNA qRT-PCR, total RNA was digested with RNase R (Epicentre, Inc.) to remove linear RNA and enrich circular RNA. The primers were listed below.

Sequences of primers in this study

| | | |
|---|---|---|
| circRNA-SORE | Forward | GTGCTCTAGGAGGTCAGTCC |
| | Reverse | GTGTGGAGTCAGAGGTACGG |
| TLE4 | Forward | GCAGAGATTGTCAAGAGGCTG |
| | Reverse | GTGATAAATGCTGGGCCTGG |
| GAPDH | Forward | CACATCGCTCAGACACCATG |
| | Reverse | TTGAGGTCAATGAAGGGGTC |
| FMRP | Forward | GTGAGGGTGAGGATTGAGGC |
| | Reverse | TGGTACCATACCCCTCTGGA |
| IMP1 | Forward | AGCTCCTTTATGCAGGCTCC |
| | Reverse | GTGTTTCGGGTGGTGCAATC |
| IMP3 | Forward | CCTGGTGAAGACTGGCTACG |
| | Reverse | ACTATCCAGCACCTCCCACT |

<u>Cell transfection</u>

Transfection of plasmids was performed using Lipofectamine® 3000. Transfections of siRNA or miRNA mimics or inhibitors (RiboBio, Guangzhou, China) or plasmids were performed using Lipofectamine® 3000 (Invitrogen, USA) according to the manufacturer's protocol.

Sequences of specific siRNA targets in this study

| | | |
|---|---|---|
| circRNA-SORE siRNA-1 | Sense (5'-3') | ACTCCATCAAGCAGCAACAdTdT |
| circRNA-SORE siRNA-2 | Sense (5'-3') | CATCAAGCAGCAACAACTCdTdT |
| FMRP siRNA | Sense (5'-3') | GGACCTTCACTCTAAGATGdTdT |
| IMP1 siRNA | Sense (5'-3') | GATCCTGGCCCATAATAACdTdT |
| IMP3 siRNA | Sense (5'-3') | CGGTGAATGAACTTCAGAAdTdT |
| YBX1 siRNA-1 | Sense (5'-3') | GTTCAATGTAAGGAACGGATdTdT |
| YBX1 siRNA-2 | Sense (5'-3') | GACGGCAATGAAGAAGATAAdTdT |
| PRP19 siRNA | Sense (5'-3') | GCCAAGTTCATCGCTTCAATTdTdT |

<u>Exosome experiments</u>

Culture medium was pre-cleared by filtration through a 0.22 μm PVDF filter (Millipore, USA), and exosomes were collected through standard centrifugation steps (twice at 110,000xg and 70 minutes). Exosomes were examined by electron microscopy using negative staining and quantified using the NanoSight NS300 instrument (Malvern Instruments Ltd. UK) equipped with NTA 3.0 analytical software (Malvern Instruments Ltd. UK). For exosomal RNA extraction, exosomes were pre-treated with RNase, and an equal number of exosomes were used for RNA extraction. For the in vitro exosome treatment, 1 μg of exosomes (equivalent to those collected from approximately 5 × 106 producer cells) were added to 2 × 105 recipient cells.

<u>*In vivo* studies</u>

For our orthotopic xenograft mice model, 16 4-6 week old male athymic BALB/c nude mice (SLAC- Shanghai Laboratory Animal Center, China) were housed and fed in standard pathogen-free conditions. Sorafenib-resistant SKhep1 cells (SKhep1-SR) were prepared as stable luciferase clones by transduction with pLKO-Luciferase lentivirus and GV248 or GV248-shcircRNA-SORE, and were then selected with G418 and puromycin to expand in culture. Intrahepatic injections of 5 × 106 cells/100 μL serum-free DMEM and matrigel (1:1) were performed on each nude mouse in groups as follows: 1) SKhep1-SR-luc; 2) SKhep1-SR-luc-shcircRNA-SORE. All surgeries were performed under sodium pentobarbital anesthesia, and all efforts were made to minimize suffering. Four weeks later, all the mice were treated with sorafenib (30 mg/kg/mouse; daily, oral gavage) as indicated for another month. Sorafenib (for groups 3 and 4) was suspended in an oral vehicle containing Cremophor (Sigma-Aldrich), 95% ethanol and water in a ratio of 1:1:6. Tumor development was monitored by IVIS once a week starting from drug treatment following intraperitoneal injection of 150mg/kg D-Luciferin. Mice were sacrificed after four weeks of treatment.

For our subcutaneous xenograft model, subcutaneous injections of 5 × 106 LM3-SR cells/100 μL serum-free DMEM and Matrigel (1:1) were performed on each nude mouse. When the xenografts reached approximately 100 mm3 (in approximately four weeks), the mice were randomized into two groups (six mice in each): 1) si-circRNA-SORE and 2) negative control. All the mice were treated with sorafenib (30 mg/kg/mouse; daily, oral gavage) and each tumor

was locally injected with 5 nmol in vivo-grade cholesterol-conjugated RIG-I siRNA for circRNA-SORE or its negative control (RiboBio, Guangzhou, China) twice a week for 2 weeks. All the mice were sacrificed after the treatment and the tumors were removed for further studies.

For our PDX models, the fragments of fresh human HCC tissues were transplanted subcutaneously into the axilla of 4- to 6-week-old NOD/SCID mice (SLAC-Shanghai Laboratory Animal Center, China). When the xenografts reached approximately 100 mm3 (approximately four weeks), all the mice were treated with sorafenib (30 mg/kg/mice; daily, oral gavage). 12 weeks later, the most resistant xenografts were isolated and mechanically disaggregated into approximately 1 mm3 tissue blocks to sub-transplant into the axilla of 4- to 6-week-old NOD/SCID mice for the second PDX generation. When the xenografts reached approximately 100 mm3 (approximately 4 weeks), the mice were randomized into two groups (six mice in each group): 1) si-circRNA-SORE and 2) negative control. All the mice were treated with sorafenib (30 mg/kg/mouse; daily, oral gavage) and each tumor was locally injected with 5 nmol in vivo-grade cholesterol-conjugated RIG-I siRNA or its negative control (RiboBio, Guangzhou, China) twice a week for two weeks. All the mice were sacrificed after the treatment and the tumors were removed for further studies.

For subcutaneous SKhep1 xenograft model, subcutaneous injections of 5 × 106 SKhep1 cells/100 μL serum-free DMEM and Matrigel (1:1) were performed on each nude mouse. When the xenografts reached approximately 100 mm3 (approximately four weeks), the mice were randomized into two groups (five for each): 1) locally injected with exosomes isolated from the media of SKhep1-SR cells and 2) locally injected with exosomes isolated from the media of SKhep1 cells. All the mice were treated with sorafenib (30 mg/kg/mouse; daily, oral gavage) and each tumor was locally injected with exosomes twice a week for totally 4 weeks. All the mice were sacrificed after the treatment and the tumors were removed for further studies.

The subcutaneous tumor size was calculated and recorded every week using the Vernier caliper as follows: tumor volume (mm3) = (L × W2)/2, where L is the long axis and W the short axis. All animal experiments were performed humanely in compliance with guidelines reviewed by the Animal Ethics Committee of the Biological Resource Centre of the Agency for Science, Technology, and Research at the Sir Run-Run Shaw Hospital, Zhejiang University School of Medicine.

H&E and immunohistochemical (IHC) staining

Tissues were fixed in 10% (v/v) formaldehyde in PBS, embedded in paraffin, and cut into 5 μm sections and used for H&E staining and IHC staining with specific primary antibodies against YBX1 (Abcam, Cambridge, MA). To enhance antigen exposure, the slides were treated with 1 × EDTA at 98 ° C for 10 minutes for antigen retrieval. The slides were incubated with endogenous peroxidase blocking solution and then were incubated with the primary antibody at 4 ℃ overnight. After rinsing with Tris-buffered saline, the slides were incubated for 45 minutes with biotin-conjugated secondary antibody, washed, and then incubated with enzyme conjugate horseradish peroxidase (HRP)-streptavidin. Freshly prepared DAB (Zymed, South San Francisco, CA) was used as a substrate to detect HRP. Finally, slides were counter-stained with hematoxylin and mounted with aqueous mounting media. Positive cells were calculated as the number of immunopositive cells × 100% divided by a total number of cells/field in 10 random fields at 400 × magnification. The IHC scoring was reviewed by two pathologists in a double blind manner. The staining results were measured semiquantitatively on a scale of (-), (+), (++)

and (+++). Representative examples of (-), (+), (++) and (+++) IHC staining for YBX1 are demonstrated in Supplementary Figure 5.

Immunofluorescence (IF) and RNA Fluorescence in situ hybridization (FISH)

Immunofluorescence was performed using a specific antibody to YBX1 (Abcam, Cambridge, MA). HepG2-SR were fixed with 4% formaldehyde (Fisher) for 15 min and then blocked with 5% normal goat serum (Vector) with or without 0.1% Triton X-100 in PBS for 60 minutes at room temperature. Immunostaining was performed using the appropriate primary and secondary antibodies. Nuclei were counterstained with DAPI.

In situ hybridization was performed using specific probes to circRNA-SORE sequence. Digoxin-labeled RNA probes were transcribed from PCR fragments using the DIG RNA labeling mix and T7 RNA polymerase (Roche) according to the manufacturers' instructions. HepG2, LM3, and SKhep1 cells were grown to the exponential phase and were 80 – 95% confluent at the time of fixation. After prehybridization (1 × PBS/0.5% Triton X-100), cells were hybridized in hybridization buffer (40% formamide, 10% Dextran sulfate, 1 × Denhardt's solution, 4 × SSC, 10mM DDT, 1mg ml-1 yeast transfer RNA, 1mg ml-1 sheared salmon sperm DNA) with DIG-labelled probes specific to circRNA-SORE at 60℃ overnight and subsequently with anti-DIG-FITC at 37℃ for 1 hour. Nuclei were counterstained with 4,6-diamidino-2-phenylindole (DAPI). The images were acquired on a TCS SP2 AOBS confocal microscope.

RNA Immunoprecipitation (RIP)

RNA immunoprecipitation was performed with the Protein A/G Agarose Beads (Santa Cruz) according to the manufacturer’s instructions. Briefly, Protein A/G Agarose Beads coated with 5 mg of normal antibodies against rabbit immunoglobulin G (Beyotime) and YBX1 (Abcam) were incubated with pre-frozen cell lysates or nuclear extracts overnight at 4℃. Associated RNA-protein complexes were collected and washed 6 times and then subjected to proteinase K digestion and RNA extraction by TRIzol. The relative interaction between protein and RNA was determined by qPCR, PCR, and normalized to input.

Mass spectrum analysis

Briefly, loaded sample onto an HPLC chromatography system named Thermo Fisher Easy-nLC 1000 equipped with a C18 column (1.8mm，0.15×1,00mm). Solvent A contained 0.1% formic acid and solvent B contained 100% acetonitrile. The elution gradient was from 4% to 18% in 182 min, 18% to 90% in 13 min solvent B at a flow rate of 300nL/min. Mass spectrometry analysis was carried out carried out in the positive-ion mode with an automated data-dependent MS/MS analysis with full scans (350-1600 m/z) acquired using FTMS at a mass resolution of 30,000 and the ten most intense precursor ions were selected for MS/MS. The MS/MS was acquired using higher-energy collision dissociation at 35% collision energy at a mass resolution of 15,000. To identify the relevant proteins, three technical replicates were performed. The intensity of each protein was obtained from three technical replicates. The criteria for the relevant proteins were as follows: the ratio of intensity between the experimental and control samples larger than 2 and p-value<0.05 was considered to be statistically significant. Those only found in a single group were also included.

PI/Annexin V apoptosis assay

Following the designated treatments, all cells including both floating and attached cells were collected by trypsinization (0.25% Trypsin, without EDTA (Gibco) and washed with PBS. The apoptotic cells were detected by Annexin V-FITC Apoptosis Detection Kit I (BD Biosciences) by staining with Annexin V-FITC and PI according to the supplier’s instructions. Viable and dead cells were detected by a BD LSRII flow cytometer (BD Biosciences).

Cell cycle analysis

Following the designated treatments, cells were trypsinized with Trypsin/EDTA (0.25%) (Gibco by Life Technologies, Grand Island, NY, USA) and then washed with PBS and fixed in ice-cold 75% ethanol overnight at −20°C. Fixed cells were washed and dissolved in RNAse and permeabilized with 0.1% Triton X-100 and subsequently stained with propidium iodide (PI) incubated at 37°C for at least 30 min. The DNA content of the cells was determined using a BD LSRII flow cytometer (BD Biosciences, Franklin Lakes, NJ, USA).

Fluorescent In Situ Detection of DNA Fragmentation (TUNEL)

Apoptotic cell death was determined by Cell-LightTM EdUTP TUNEL Cell Detection Kit (RiboBio, Guangzhou, China), following the manufacturer's protocol. Morphological changes in HCC cells undergoing apoptosis were then detected by counterstaining with DAPI. The slides were examined by fluorescence microscopy.

Statistical Analysis

Data are expressed as mean ± SEM from at least three independent experiments. Statistical analyses used Student’s t-test, Chi-square test, Kaplan-Meier survival analysis, log-rank test and Cox regression analysis with GraphPad Prism 8.0 (GraphPad Software, Inc., La Jolla, CA). $P < 0.05$ was considered statistically significant. $P > 0.2$ was used for exclusion in multivariate analyses. Gene Ontology Analysis was performed online by Database for Annotation, Visualization and Integrated Discovery (DAVID) bioinformatics resources (v6.8)[1, 2].

**Supplementary Figure Legends**

Figure S1: CircRNA-SORE is critical for maintaining sorafenib resistance

(A) Left: qPCR analysis of circRNA_SORE expression after transfecting HepG2-SR cells with si-NC, si-circRNA-SORE-1, and si-circRNA-SORE -2. Right: schematic representation of where the siRNAs were designed. The siRNA sequences are shown below. (B) Cell viability after transfecting HepG2-SR cells with si-NC, si-circRNA-SORE-1 or si-circRNA-SORE-2. (C) qPCR analysis of circRNA-SORE expression after transfecting sorafenib-resistant cells with si-circRNA_104797. (D) Brightfield images demonstrating cell morphology of the three parental cell lines with or without adding si-circRNA-SORE after sorafenib treatment at the indicated concentrations and hours. Scale bar, 20μm. (E) TUNEL (green) staining of sorafenib-resistant cells transfected with si-NC and si-circRNA-SORE. The nucleus was shown in blue. Scale bar, 50μm. (F) qPCR analysis of circRNA-SORE expression after 72-hour of sorafenib treatment in parental HCC cells overexpressed with cricRNA-SORE. (G) Relative cell viability after 72-hour sorafenib treatment in parental HCC cells overexpressed with circRNA-SORE. (H) Images of colony formation assay wells after 72-hour sorafenib treatment in parental HCC cells overexpressed with circRNA-SORE. (I) PI/Annexin V flow cytometry analysis of parental HCC cells overexpressed with NC or circRNA-SORE. Three different independent experiments with three technical repetitions were performed. Data are expressed as mean ± SEM. Statistical analyses used Student's t-test, $p<0.05$ was considered statistically significant. *** $p<0.001$.

Figure S2: CircRNA-SORE functions by binding to YBX1 protein

(A) qPCR analysis of TLE4 mRNA expression in HCC parental (P) and sorafenib-resistant (R) cell lines. (B) qPCR analysis of TLE4 mRNA expression in HCC sorafenib-resistant cell lines with or without si-circRNA-SORE. (C) Western blot analysis for TLE4 and β-tubulin in HCC sorafenib-resistant cell lines with or without si-circRNA-SORE. (D) Western blot analysis for eIF4A3, FMRP, IMP1 and IMP3 in circRNA-SORE pull-down lysates of HepG2-SR cells. (E) qPCR evaluation of mRNA isolated from HepG2-SR cells that were transfected with FMRP, IMP1, and IMP3 siRNA. (F) Western blot analysis for FMRP, IMP1, and IMP3 in their siRNA-transfected HepG2-SR cells. (G) Relative cell viability of HepG2-SR cells transfected with si-FMRP, si-IMP1 and si-IMP3 in the treatment of sorafenib. (H) Gene Ontology Analysis of the 129 up-regulated and 301 down-regulated proteins (FC>2 and $P<0.05$) in the circRNA-SORE silenced group compared to the control group in HepG2-SR cells. C: Cellular Component; P: Biological Process; F: Molecular Function. (I) The spectrogram of YBX1 from LC-MS/MS. (J) Schematic illustration of the biotin pull-down assay for YBX1 using Western blot and qPCR analysis for circRNA-SORE in HepG2-SR cells (left). Schematic illustration of the reciprocal RNA immunoprecipitation using YBX1 for circRNA-SORE in HepG2-SR cells (right). (K) Box-plot of YBX1 expression in TCGA HCC tumor and matched TCGA normal liver tissues along with GTEx data. (L) Gene Expression Profile of YBX1 expression in TCGA HCC tumor and matched TCGA normal liver tissues along with GTEx data. (M) Brightfield images of sorafenib-resistant cells following si-YBX1-2 transfection and sorafenib treatment (7μM for HepG2-SR, 6μM for LM3-SR and SKhep1-SR) for 48 h. Scale bar, 50 μm. (N) Western blot analysis of YBX1 and β-tubulin in HepG2-P cells overexpressing (OE) circRNA-SORE by lentivirus and transfected with MAO-YBX1. (O) Representative examples of (-), (+), (++) and (+++) IHC staining for YBX1 (Scale bar, 50 μm). Three different independent experiments with three technical repetitions were performed. Data are expressed as mean ± SEM. Statistical analyses

used Student's t-test, and $p<0.05$ was considered statistically significant. *** $p<0.001$. NS, not significant.

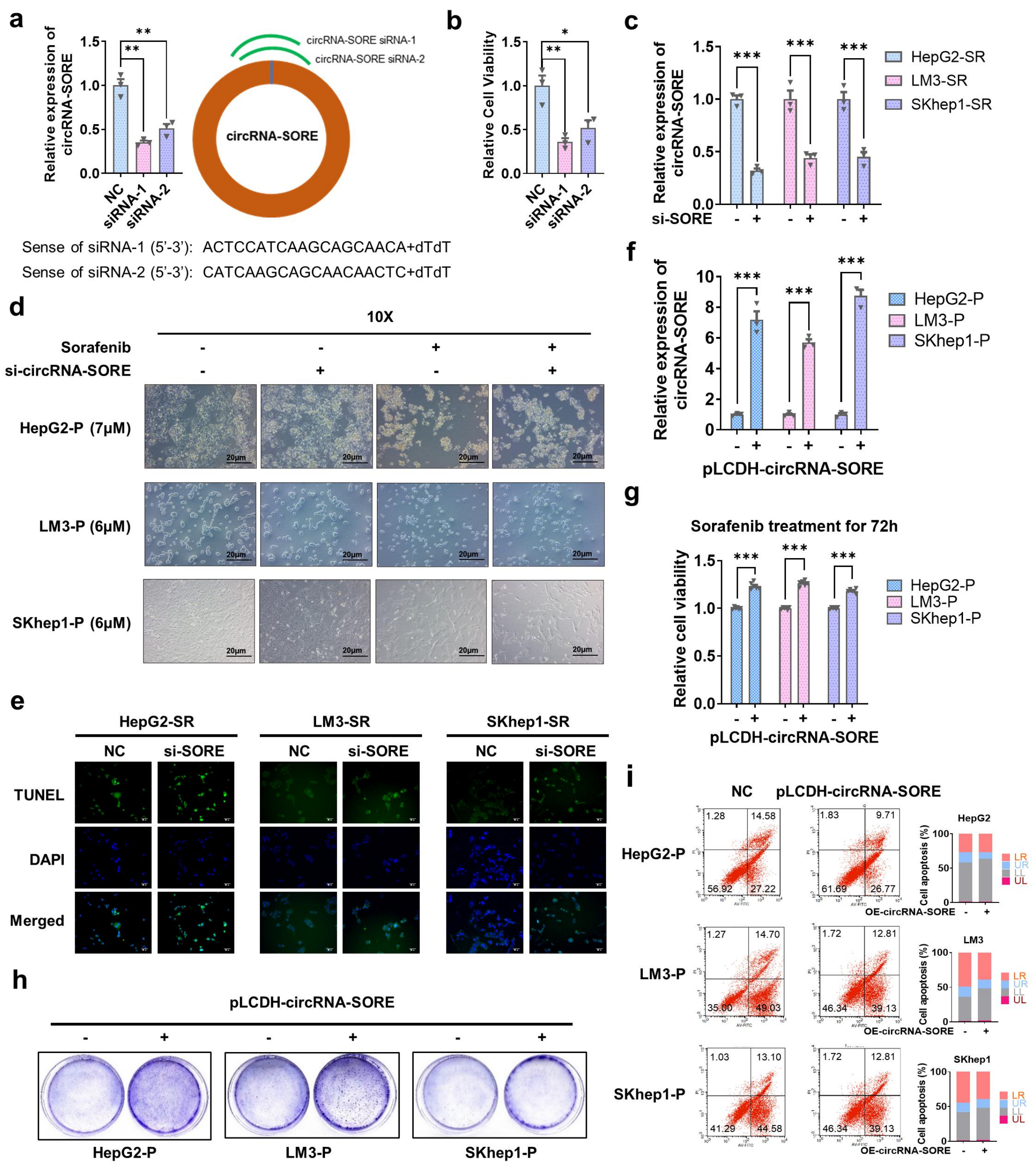


## Supplementary Figure 1

CircRNA-SORE is critical for maintaining sorafenib resistance.

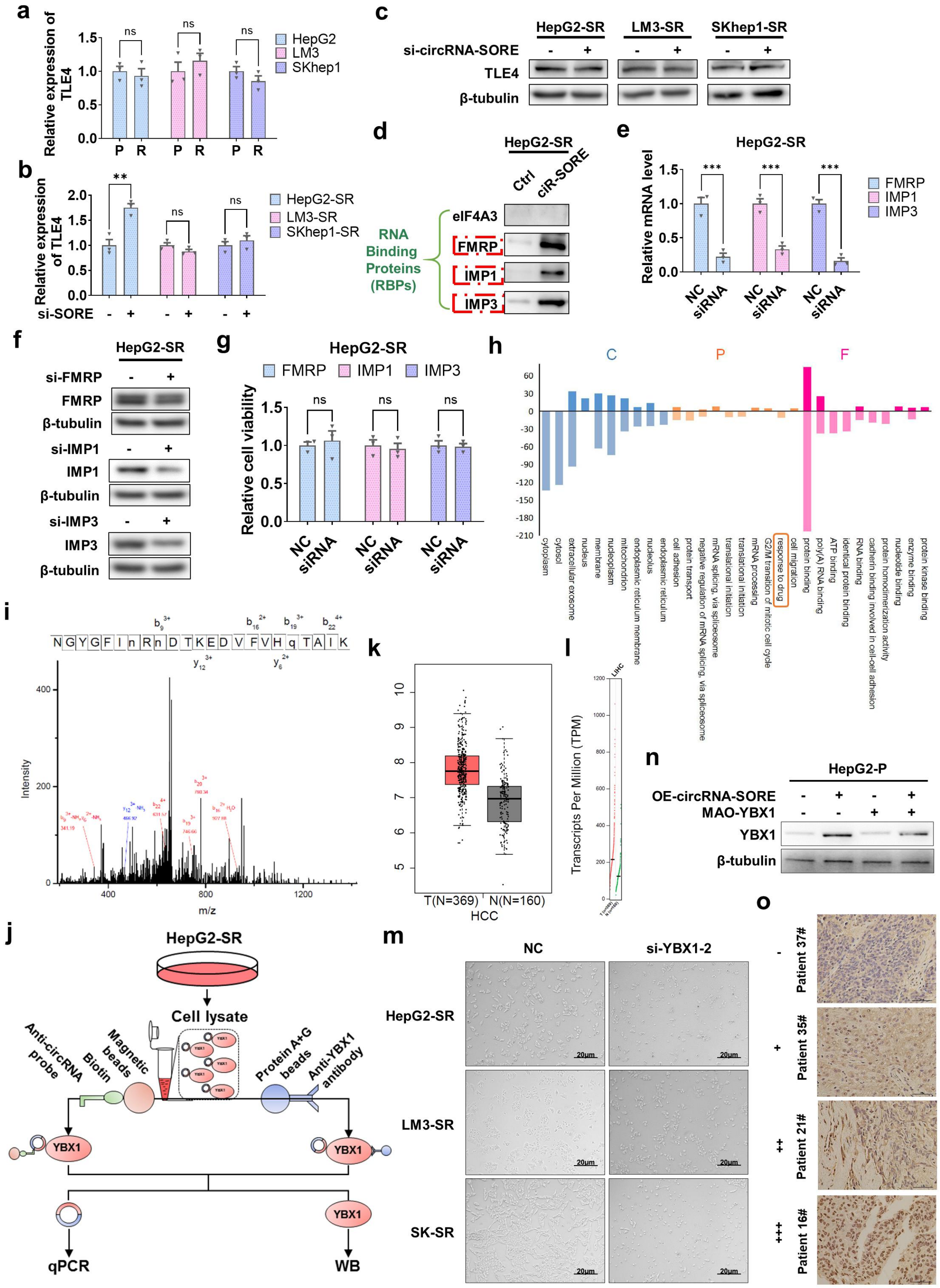


**Supplementary Figure 2**

CircRNA-SORE functions by binding to YBX1 protein.

**Supplementary Table 1.**

**The sequence of wildtype and mutant of circRNA-SORE**

Wildtype of circRNA-SORE:

CAGCAACAACTCCAGGCCCAGCATTTATCACATGGACATGGTCTCCCCGTACCTCTG
ACTCCACACCCTTCAGGGCTCCAGCCCCCTGCCATTCCACCCATCGGTAGCAGTGCC
GGGCTTCTGGCCCTCTCCAGTGCTCTAGGAGGTCAGTCCCATCTTCCAATTAAAGAT
GAGAAGAAGCACCATGACAATGATCACCAAAGAGACAGAGACTCCATCAAG

Bold: the wildtype YBX1 binding motif

Mutation of circRNA-SORE:

CAGCAACAACTCCAGGCCCAGCATTTATCACATGGACATGGTCTCCCCGTACCTCTG
ACTCCACACCCTTCAGGGCTCCAGCCCCCTGCCATTCCACCCATCGGTAGCAGTGCC
GGGCTTCTGGCCCTCTCCAGTGCTCTAGGAGGTCAGTCCCATCTTTACGCTAAAGAT
GAGAAGAAGCACCATGACAATGATCACCAAAGAGACAGAGACTCCATCAAG

Bold: the mutant YBX1 binding motif

**Supplementary Table 2.**

The list of proteins that could both specifically bind to and be regulated by circRNA-SORE.

| Gene name | Direction |
|---|---|
| Y-box binding protein 1(YBX1) | Down |
| heterogeneous nuclear ribonucleoprotein U like 1(HNRNPUL1) | Down |
| histone cluster 1 H4 family member i(HIST1H4I) | Up |
| karyopherin subunit alpha 3(KPNA3) | Down |
| keratinocyte proline rich protein(KPRP) | Up |
| methionine adenosyltransferase 1A(MAT1A) | Down |
| phosphoserine phosphatase(PSPH) | Down |
| spectrin repeat containing nuclear envelope protein 2(SYNE2) | Down |
| transferrin(TF) | Down |
| ubiquilin 1(UBQLN1) | Down |

**Supplementary Table 3**.
Results of univariate and multivariate analysis of clinicopathological factors for overall-survival (Cox proportional hazard model)

| Variables | Univariate analysis | | | Multivariate analysis (YBX1) | | |
|---|---|---|---|---|---|---|
| | HR | 95% CI | P value | HR | 95% CI | P value |
| Age (Old/Young) | 0.975 | 0.936-1.015 | 0.217 | 2.955 | 0.712-12.262 | 0.136 |
| Gender (Female/Male) | 0.830 | 0.109-6.330 | 0.858 | 0.257 | 0.026-2.557 | 0.246 |
| HBV (+/-) | 1.227 | 0.413-3.650 | 0.713 | | | |
| Fibrosis (+/-) | 3.212 | 0.723-14.271 | 0.125 | 3.699 | 0.720-19.012 | 0.117 |
| Stage* (Late/ Early) | 1.557 | 0.489-4.957 | 0.454 | | | |
| Micro-vascular invasion (Present/Absent) | 1.680 | 0.606-4.661 | 0.319 | 2.809 | 0.879-8.978 | 0.082 |
| YBX1** (++/+++vs.-/+) | 5.208 | 1.789-15.161 | 0.002 | 10.797 | 2.929-39.799 | 0.0004 |

*Bureau of Medical Administration, National Health and Family Planning Commission of the People's Republic of China. Standardization of diagnosis and treatment for hepatocellular carcinoma (2017 edition). Chinese Journal of Digestive Surgery 16, 635-647.
** YBX1 levels were defined by IHC staining.
As a positive correlation of YBX1 protein level and circRNA-SORE expression level was observed in Table 1, to avoid interations between factors, the multivariate analyses were performed respectively for circRNA-SORE and YBX1.

**References**

1. Huang da W, Sherman BT, Lempicki RA. Systematic and integrative analysis of large gene lists using DAVID bioinformatics resources. Nat Protoc 2009;4:44-57.
2. Huang da W, Sherman BT, Lempicki RA. Bioinformatics enrichment tools: paths toward the comprehensive functional analysis of large gene lists. Nucleic Acids Res 2009;37:1-13.
